# A novel ten-gene prognostic signature for cervical cancer based on CD79B-related immunomodulators

**DOI:** 10.3389/fgene.2022.933798

**Published:** 2022-11-02

**Authors:** Dan Pu, Dan Liu, Can Li, Chunyan Chen, Yuxin Che, Jiaoyan Lv, Yang Yang, Xuelian Wang

**Affiliations:** ^1^ Department of Microbiology and Parasitology, College of Basic Medical Sciences, China Medical University, Shenyang, China; ^2^ Department of Gynecology, The Fourth Affiliated Hospital of China Medical University, Shenyang, China; ^3^ Department of Medical Basic Experimental Teaching Center, China Medical University, Shenyang, China

**Keywords:** CD79B, cervical cancer, tumor microenvironment, prognosis, tumor-infiltrating immune cells

## Abstract

The identification of immune-related prognostic biomarkers opens up the possibility of developing new immunotherapy strategies against tumors. In this study, we investigated immune-related biomarkers in the tumor microenvironment to predict the prognosis of cervical cancer (CC) patients. ESTIMATE and CIBERSORT algorithms were used to calculate the abundance of tumor-infiltrating immune cells (TICs) and the amount of immune and stromal components in cervical samples (*n* = 309) from The Cancer Genome Atlas. Ten immune-related differentially expressed genes associated with CC survival were identified *via* intersection analyses of multivariate Cox regression and protein-protein interactions. CD79B was chosen for further study, and its prognostic value and role in anti-CC immune functions were analyzed. Differential expression analysis and qRT-PCR validation both revealed that CD79B expression was down-regulated in CC tissues. Survival analysis suggested that a high level of CD79B expression was associated with good prognosis. In the clinical correlation analysis, CD79B expression was found to be related to primary therapy outcome, race, histological type, degree of cell differentiation, disease-specific survival, and progression-free interval. GSEA showed that the function and pathway of CD79B were mainly related to immune activities. Meanwhile, CD79B expression was correlated with 10 types of TICs. Based on CD79B-associated immunomodulators, a novel immune prognostic signature consisting of 10 genes (CD96, LAG3, PDCD1, TIGIT, CD27, KLRK1, LTA, PVR, TNFRSF13C, and TNFRSF17) was established and validated as possessing good independent prognostic value for CC patients. Finally, a nomogram to predict personalized 3- and 5-year overall survival probabilities in CC patients was built and validated. In summary, our findings demonstrated that CD79B might be a potential prognostic biomarker for CC. The 10-gene prognostic signature independently predicted the overall survival of patients with CC, which could improve individualized treatment and aid clinical decision-making.

## 1 Introduction

Cervical cancer (CC) is the fourth most common cancer in women (Arbyn et al., 2020), and its occurrence is associated with persistent infection with high-risk human papillomavirus (HPV) (Cohen et al., 2019). Approximately 90% of cervical cancer deaths occur in developing regions of the world (Torre et al., 2015). In recent years, substantial progress has been made in controlling cervical cancer due to widespread screening and extensive vaccination against HPV infection (Ventriglia et al., 2017). However, recurrent and advanced-stage disease is not amenable to radical treatment, and *de novo* metastatic disease is still considered incurable, with poor prognosis (Ventriglia et al., 2017; Liontos et al., 2019). Fortunately, immunotherapy is emerging as a potential novel therapeutic approach to improving outcomes in CC patients (Dyer et al., 2019).Biomarkers associated with cancer may represent critical targets for improving cancer therapies (Liu, 2019). Thus, there is an urgent need to investigate novel immune-related biomarkers in CC to develop new immunotherapy strategies.

Over the past few decades, immunotherapy has become a powerful clinical strategy in the treatment of cancer (Riley et al., 2019). It works primarily by harnessing an anti-tumor immune response (Sanmamed and Chen, 2018). Today, there are many types of tumor immunotherapies, such as checkpoint inhibitors, lymphocyte-promoting cytokines, cancer vaccines, oncolytic viruses, and bispecific antibodies (Riley et al., 2019). Immunotherapy with immune checkpoint inhibitors, especially those that target the programmed death-ligand 1/programmed death-1 (PD-L1/PD-1) and cytotoxic T-lymphocyte-associated antigen-4 (CTLA-4) pathway, has improved the effects of treatment on various types of tumors (Apolo et al., 2017). In recent years, anti-PD-1/PD-L1 checkpoint blockade immunotherapy (CBI) has been approved to treat metastatic squamous cell carcinomas, including head and neck squamous cell carcinoma, lung cancer, and cervical cancer (Lyu et al., 2020). It has been proven that PD-1/PD-L1 inhibitors benefit cervical cancer treatment by markedly reinvigorating the anti-tumor immune response of T cells (Wang et al., 2019; Balanca et al., 2020; Lyu et al., 2020). However, the sustained therapeutic effect of anti-PD-L1 treatment alone on CC was limited (Liu et al., 2016).

The effectiveness of tumor immunotherapy is largely influenced by the tumor microenvironment (TME) (Binnewies et al., 2018). The TME is a complex ecosystem comprising tumor cells, immune cells, stromal cells, abnormal vasculature, chemokines, and cytokines, which may have an effect on tumor occurrence, progression, and metastasis (Fridman et al., 2017; Wu et al., 2019). The TME carries out essential functions that aid the tumor in tolerating immune surveillance (Qiu et al., 2020). In cervical cancer, it influences prognosis, with higher ratios of tumor-infiltrating CD8^+^ T cells being associated with improved survival (Otter et al., 2019). An in-depth analysis of the complexity of the tumor immune microenvironment in cervical cancer may therefore reveal biomarkers that will help identify novel targets for immunotherapeutic regulation (Binnewies et al., 2018).

CD79B is aB-cell receptor–associated protein, physiologically expressed inB cells and mostB-cell malignancies (Visco et al., 2020). According to three recent studies,B cells are vital immune components within tumors and are associated with immunotherapy outcomes (Bruno, 2020). At present, antibody-drug conjugates targeting the pan–B cell biomarker of CD79B have been proven effective in clinical applications for hematological malignancies, such as different subtypes of molecular diffuse largeB-cell lymphoma (Pfeifer et al., 2015; Bruno, 2020; Harris et al., 2020). However, the function of CD79B in CC has not yet been investigated.

In this study, we explored the role of CD79B in anti-CC immune function and its potential as a prognostic marker for CC. We conducted multiple bioinformatics analyses, starting from the differentially expressed genes (DEGs) generated by comparing the immune and stromal components of cervical samples in the Cervical Squamous Cell Carcinoma and Endocervical Adenocarcinoma (CESC) dataset of The Cancer Genome Atlas (TCGA). Furthermore, we systematically evaluated the correlation between CD79B and immune cell infiltration, as well as the signaling pathways regulating the CD79B-mediated immune response. Finally, we used CD79B-associated immunomodulators to create the immune prognostic signature, and we constructed a nomogram by integrating the risk score in the signature and other clinical characteristics.

## 2 Methods and materials

### 2.1 Raw data preparation and workflow

**FIGURE 1:**
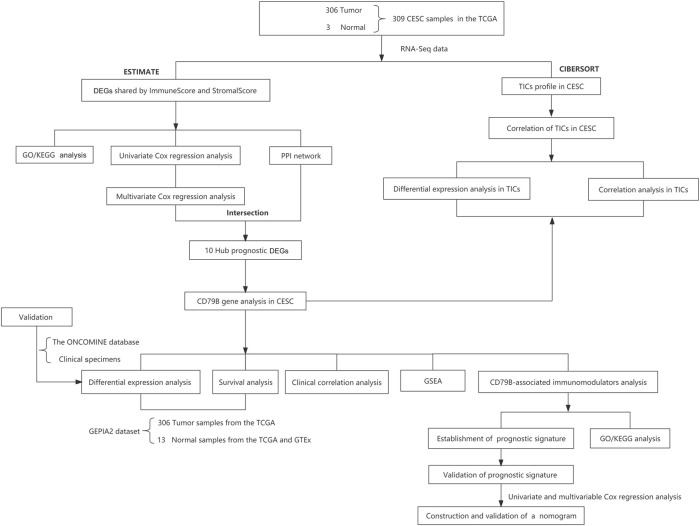
Study design and workflow.

Transcriptome RNA sequencing (RNA-seq) data (HTSeq-FPKM) of 309 cervical samples, including 306 cancerous and three normal samples, and the data of the 306 corresponding clinical cases (Supplementary Table S1), was downloaded from the TCGA portal maintained by GDC (https://portal.gdc.cancer.gov/; up to 4 July 2021). The limma package of R software was used to further process the RNA expression data (Ritchie et al., 2015). Meanwhile, we used the RNA sequencing expression data from the Gene Expression Profiling Interactive Analysis 2 (GEPIA2) database (http://gepia2.cancerpku.cn/#index) to analyze the differential expression of CD79B between cervical tissues and normal tissues, as well as the link between CD79B expression and CC patient survival. The GEPIA2 dataset for analysis included 13 normal samples from the TCGA and the Genotype-Tissue Expression (GTEx) database (https://gtexportal.org/home/), respectively, as well as 306 tumor samples from the TCGA database. Furthermore, after determining the differential expression of CD79B in CC using the GEPIA2 data, we validated this determination using 30 cervical tissue samples from the local hospital and the gene expression array dataset from the ONCOMINE database (https://www.oncomine.org). This validation dataset included 28 cervical samples (20 cancerous samples and eight normal samples). The workflow of our study is shown in Figure 1.

### 2.2 Generation of immunescore, stromalscore, and ESTIMATEScore

Infiltrating stromal and immune cells form a significant part of the tumor tissue and play vital roles in cancer biology (Yoshihara et al., 2013). Here, we used the ESTIMATE algorithm in the R language and loaded the “Estimate” R package to evaluate the proportion of immune matrix components of each transcriptome data sample in the TME, including ImmuneScore, StromalScore, and ESTIMATEScore.

### 2.3 Identification and enrichment analysis of differentially expressed genes

According to the comparison of the median StromalScore and ImmuneScore values, 306 CC samples were labeled as high-scoring or low-scoring, respectively. A differential expression analysis of these genes was performed using the “limma” R package, and DEGs were obtained by comparing high-scoring samples with low-scoring samples. A false discovery rate (FDR) adjusted *p*-value < 0.05 and |log2 (fold change)| > 1 were considered statistically significant. The results were plotted using the “heatmap” package in R. Finally, to better understand the function of the DEGs shared by ImmuneScore and StromalScore, pathway enrichment analyses were performed on Gene Ontology (GO) (Subramanian et al., 2005) and Kyoto Encyclopedia of Genes and Genomes (KEGG) (Kanehisa et al., 2017) data using the “clusterProfiler, enrichplot, and ggplot2” package in R software. GO and KEGG terms with *p*- and *q*-values < 0.05 were considered significantly enriched. The top five results of the enrichment analysis were visualized *via* bubble charts.

### 2.4 Intersection analysis of the PPI network and multivariate cox regression

Analysis of protein-protein interaction (PPI) is a powerful method for characterizing and inferring the potential interactions between proteins (Goehler et al., 2004). In this study, based on the TCGA data, the PPI network of the DEGs was constructed using the STRING database (https://string-db.org/cgi/input.pl), then visualized with Cytoscape software (version 3.6.1). Nodes whose interactive relationships had a confidence interval greater than 0.95 were used for building networks. Meanwhile, the top 25 DEGs were selected to create bar plots according to the number of nodes. Univariate and multivariate Cox regression analyses using R software with the “survival” package were applied to evaluate the DEGs’ prognostic values in CC. Statistical significance was defined as a *p*-value < 0.05 in the univariate Cox regression analysis and a *p*-value < 0.001 in the multivariate Cox regression analysis. Consequently, the top 25 DEGs in the PPI network were intersected with the most significant factors obtained from the multivariate Cox regression analysis.

### 2.5 Differential expression analysis of CD79B based on the qRT-PCR experiment and online databases

We analyzed the CESC dataset from the GEPIA2 database for the differential expression analysis of CD79B in normal and cancerous cervical tissues. Meanwhile, to validate the differential expression of CD79B from the GEPIA2 analysis, we analyzed the dataset in the ONCOMINE database and conducted a quantitative real-time reverse transcription polymerase chain reaction (qRT-PCR) experiment using 30 clinical samples (15 cancerous samples; 15 normal samples) from CC patients. For the qRT-PCR experiment, total RNA was isolated from 30 samples of cervical cancer and normal tissues using Trizol reagent (Takara, Dalian, China) according to the manufacturer’s instructions. The extracted RNA was reverse transcribed into cDNA using a Reverse Transcription Kit (Takara). SYBR Green qPCR Master Mix (Vazyme, Nanjing, China) was applied to perform the qRT-PCR analysis. Primer sequences for CD79B and *β*-actin (the internal reference gene) were as follows: CD79B (forward primer: 5′-GGG​CTG​GAG​ACA​AAT​GGC​AG-3′; reverse primer: 5′- TGA​AGT​GGT​CTG​TAG​GTG​AGC​A-3′); *β*-actin (forward primer: 5′-ATG​TGG​CCG​AGG​ACT​TTG​ATT-3′; reverse primer: 5′-AGT​GGG​GTG​GCT​TTT​AGG​ATG-3′). The CD79B gene expression value was normalized to the expression value of the *β*-actin gene. Relative mRNA expression levels were calculated using the 2^−ΔΔCt^ method, and the data result was analyzed by unpaired *t*-test with Welch’s correction using SPSS Statistics software.

### 2.6 Survival analysis and clinical correlation analysis of CD79B

We analyzed the effect of CD79 B on survival in CC patients using the CESC dataset on the GEPIA2 database. Moreover, to further explore the correlation between CD79B expression and clinicopathological characteristics, the clinical data and CD79B expression data of 306 CESC samples were obtained from the TCGA database. In the analysis of these data, the CD79B expression levels were respectively divided into high and low groups based on their median levels. The Wilcoxon rank-sum test was used to analyze the differences for continuous variables. For categorical variables, the Fisher’s exact test or chi-squared test was used to differentiate the rates of different groups. Statistical significance was considered at *p* < 0.05.

### 2.7 Gene set enrichment analysis

To explore the biological functions and signaling pathways of the CD79B gene, we used Gene Set Enrichment Analysis (GSEA) embodied in a freely available software package (version 4.0.3) (Subramanian et al., 2005) to identify enriched GO terms and KEGG pathways associated with high CD79B expression. The number of random sample alignments was set at 1,000. Gene sets with | NES | ≥ 1, NOM *p*-value < 0.001, and FDR q-value < 0.001 were defined as statistically significant.

### 2.8 Analysis between CD79B and tumor-infiltrating immune cells

The CIBERSORT algorithm was applied to characterize the cell composition of complex tissues according to their gene expression profiles and to estimate the profile and abundance of tumor-infiltrating immune cells (TICs) in all tumor samples with immune infiltration scores. CIBERSORT can thus be used to perform large-scale RNA mixtures analysis to find therapeutic targets and cellular biomarkers (Newman et al., 2015). In this study, the CIBERSORT R script (https://cibersort.stanford.edu/) was applied to qualify and quantify 22 types of immune cells (seven T cell types, naïve and memoryB cells, plasma cells, NK cells, and myeloid subsets) in cervical tissues. After excluding samples with *p* ≥ 0.05, the remaining samples were selected for further analysis. The results were visualized using bar charts, corr plots, violin plots, and heatmaps, respectively, by the corresponding R packages.

### 2.9 Analysis of CD79B-associated immunomodulators

Multiple types of data resources in oncoimmunology, such as lymphocytes, immunomodulators, and chemokines, were available on the TISIDB online platform (http://cis.hku.hk/TISIDB/). The TISIDB online tool can be used to comprehensively investigate the interactions between the tumor and immune cells (Ru et al., 2019). To analyze the correlation between CD79B expression and immunomodulators, we extracted 45 immunostimulators and 24 immunoinhibitors from the TISIDB online portal. Additionally, 57 immunomodulators that were significantly correlated with CD79B expression (per the Spearman correlation test, *p* < 0.05) were chosen for analysis. Out of these, 52 immunomodulators that were highly likely to be actively involved were used to establish a PPI network *via* the STRING database and visualized by Cytoscape software. At the same time, KEGG pathway enrichment and GO analyses for these 52 CD79B-related immunomodulators were performed using WebGestalt (http://www.webgestalt.org), a gene set enrichment analysis tool (Zhang et al., 2005).

### 2.10 Establishment of the immunomodulator prognostic signature and survival analysis

Based on the CD79B-related immunomodulators, we attempted to develop a multiple immune gene signature to predict the prognosis of CC patients. The Akaike Information Criterion in the Cox models was used for the stepwise variable selection (Choi et al., 2011). After screening the immune genes, we calculated the risk score of the immune gene signature in each CESC patient *via* the following formula: risk score = [Expression level of Gene A × coefficient] + [Expression level of GeneB × coefficient] + …+ [Expression level of Gene N × coefficient](Dai et al., 2021). According to the prognostic model, we calculated the risk score of each cervical cancer patient and used the medium value of the risk score to divide the patients into a high-risk group and a low-risk group. At the same time, we also drew the Kaplan–Meier survival curve and the receiver operating characteristic (ROC) curve to evaluate the signature’s prediction accuracy. Additionally, to assess whether the immune-related gene signature has an independent prognostic value, univariate and multivariate Cox regression analysis was performed for the risk score, with adjustments for age, stage, histological type, and body mass index (BMI).

### 2.11 Construction and validation of the nomogram

Nomograms are widely used to estimate cancer prognosis or other clinical outcomes because they can simplify statistical predictive models into a single numerical estimate of the probability of an event (Iasonos et al., 2008). We therefore plotted the nomogram, according to clinical characteristics and risk score, *via* the “rms” R package to predict the probability of three- and five- year overall survival (OS) for CC patients. To measure the predictive accuracy of the nomogram model, the concordance index (C-index) was calculated. Then, the discriminative ability of the nomogram was determined by a calibration curve using the bootstrap method (1,000 replicates) to test the reliability between the predicted and actual OS rates (Wang et al., 2013). All analyses were performed using R software.

### 2.12 Statistical analysis

Statistical analysis was performed using R software (version 4.0.4) and complemented by IBM SPSS Statistics 23.0. The threshold of statistical significance was set at *p* < 0.05 (*p < 0.05).

## 3 Results

### 3.1 Identification and enrichment analysis of DEGs shared by immunescore and stromalscore

**FIGURE 2:**
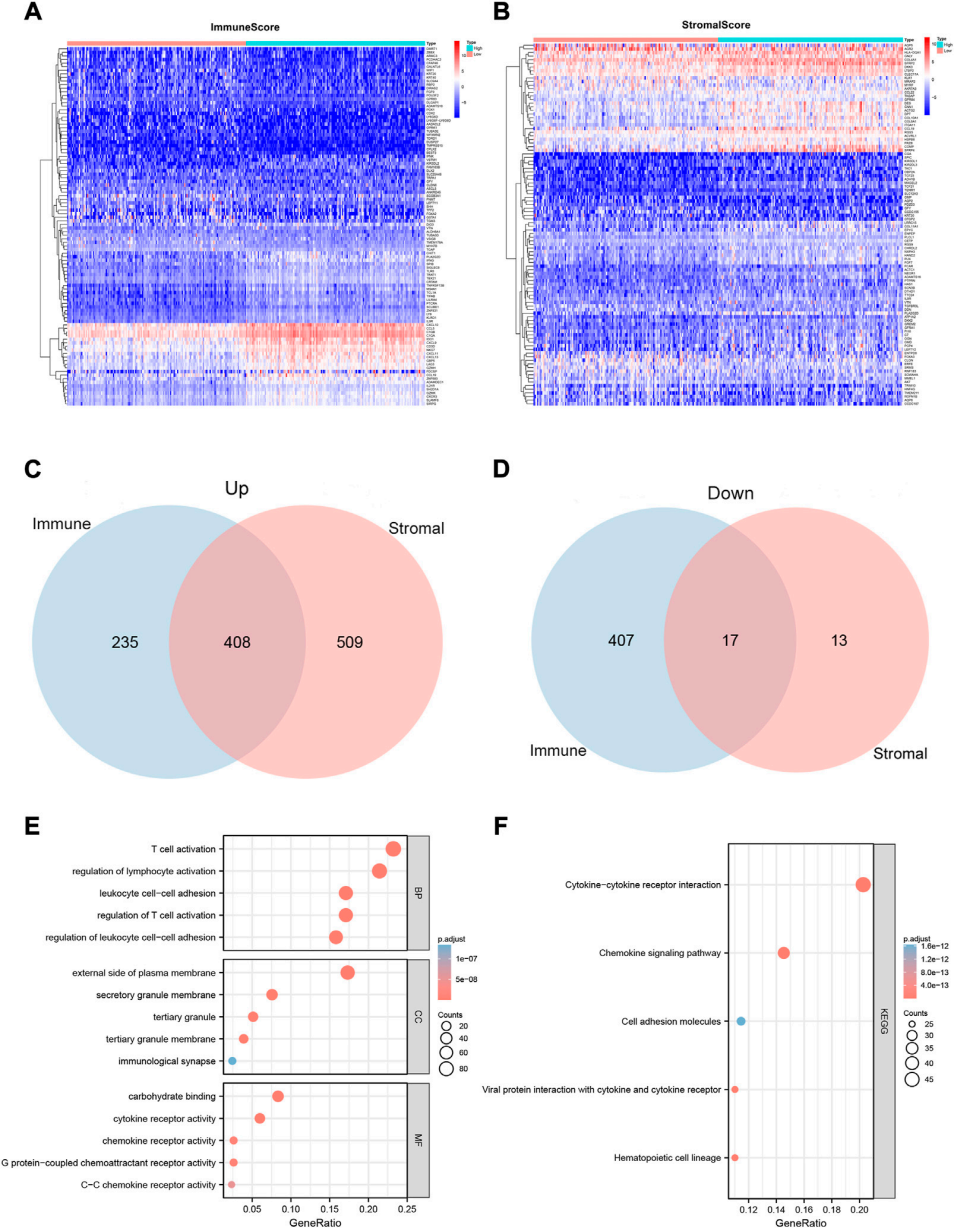
Heatmaps, Venn plots, and enrichment analysis of GO and KEGG terms for the CESC dataset. **(A and B)** Heatmaps for the DEGs obtained from ImmuneScore **(A)** and StromalScore **(B)**, generated by comparing high-scoring and low-scoring groups of immune and stromal cells. **(C and D)** Venn diagram analysis of up-regulated **(C)** and down-regulated **(D)** DEGs shared by the ImmuneScore and StromalScore. **(E and F)**Biological functions **(E)** and pathway **(F)** enrichment analyses for DEGs in the CESC dataset with *p*-value and *q*-value < 0.05.

To determine the exact alterations of the gene profiles in the immune and stromal components, we performed a comparative analysis of the gene expression in samples with high and low ImmuneScore/StromalScore values. A total of 1,067 DEGs were obtained from ImmuneScore, comprising 643 up-regulated and 424 down-regulated genes (Figure 2A). Similarly, 947 DEGs were obtained from StromalScore, comprising 917 up-regulated and 30 down-regulated genes (Figure 2B). The Venn diagram results, obtained by intersecting the DEGs from ImmuneScore and StromalScore, indicated that 408 genes were up-regulated and 17 genes were down-regulated (Figures 2C,D).

We also predicted the functions of 425 intersecting DEGs. The results of the GO enrichment analysis showed that these DEGs were mainly involved in immune-related GO terms, such as T cell activation, regulation of lymphocyte activation, and regulation of T cell activation (Figure 2E). KEGG enrichment analysis revealed that these DEGs were significantly enriched in cytokine–cytokine receptor interaction, hematopoietic cell lineage, viral protein interaction with cytokines and cytokine receptors, chemokine signaling pathways, and cell adhesion molecules (Figure 2F). Thus, the overall function of 425 DEGs was focused on immune-related activities, which suggests that the involvement of immune factors and components plays a crucial role in the TME status of CC patients.

### 3.2 Intersection analysis of the PPI network and Multivariate Cox regression

**FIGURE 3:**
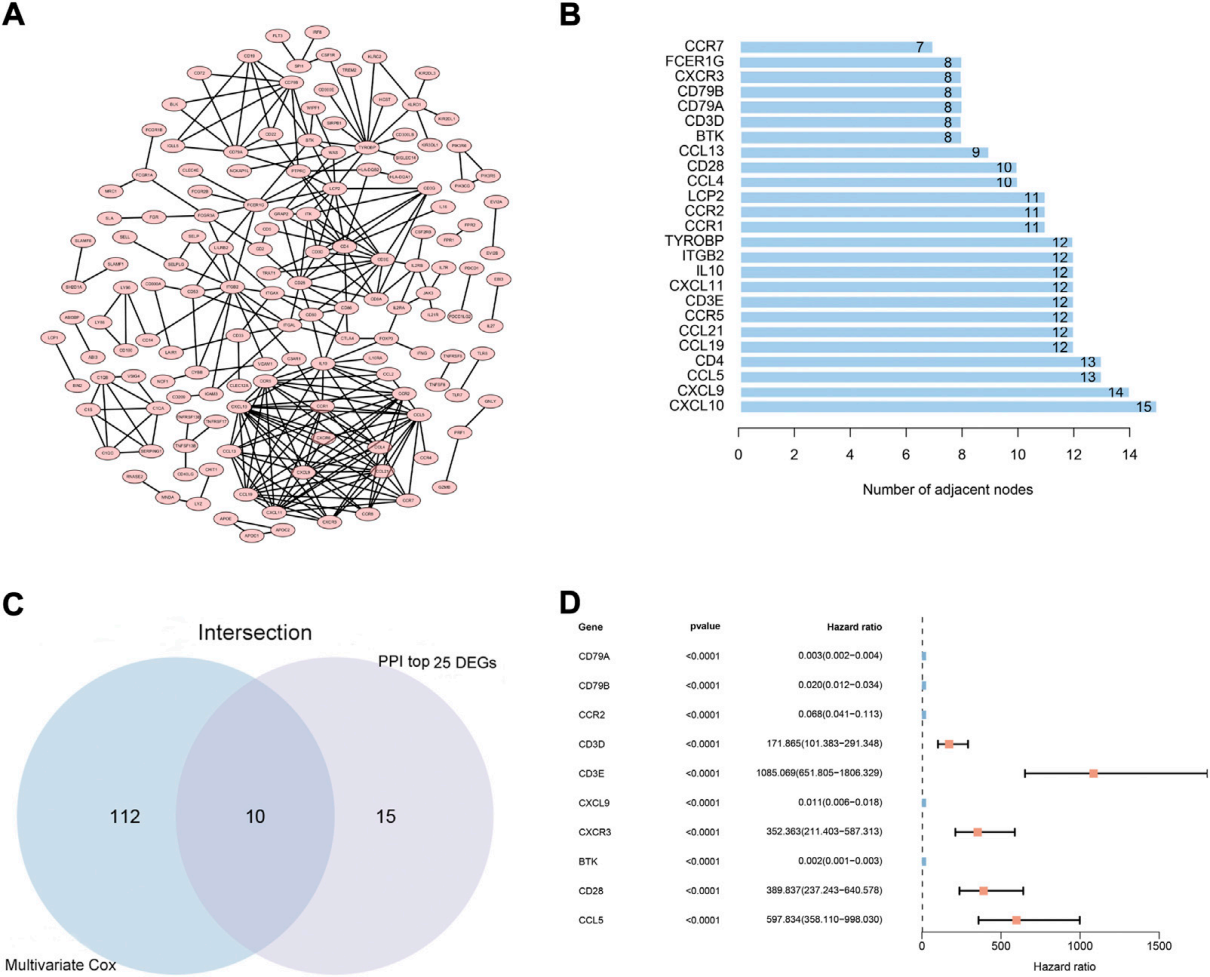
PPI network and multivariate Cox regression analysis. **(A)** PPI network constructed using nodes with interaction confidence value >0.95, as visualized by Cytoscape. **(B)** The top 25 DEGs, ordered by the number of nodes. **(C)** Venn diagram of the common intersection DEGs based on the top 25 DEGs in the PPI network and the most significant DEGs with *p* < 0.001 in multivariate Cox regression analysis. **(D)** Multivariate Cox regression analysis of 10 common intersecting DEGs.

To explore the possible mechanisms underlying the 425 DEGs, a PPI network based on the STRING platform was constructed. The 146 DEGs in the PPI network with a high likelihood of interaction (score > 0.95) are shown in Figure 3A, and the top 25 DEGs selected using the number of nodes and edges in the PPI network are shown in Figure 3B. Univariate Cox regression analysis on the 425 DEGs showed that 146 of them were considered statistically significant (*p* < 0.05) (Supplementary Table S2). The multivariate Cox regression analysis, based on the 146 DEGs in the univariate Cox regression analysis, showed that 122 DEGs were considered statistically significant for the survival of CC patients (*p* < 0.001) (Supplementary Table S3). Ultimately, 10 DEGs (CCL5, CD3E, CXCL9, CD28,BTK, CD3D, CD79A, CD79B, CXCR3, CCR2) (Figures 3C,D) were identified based on the intersection of the top 25 DEGs in the PPI network and the 122 significant DEGs in the multivariate Cox regression analysis.

### 3.3 Differential expression of CD79B, survival, and clinical correlation analysis

**FIGURE 4:**
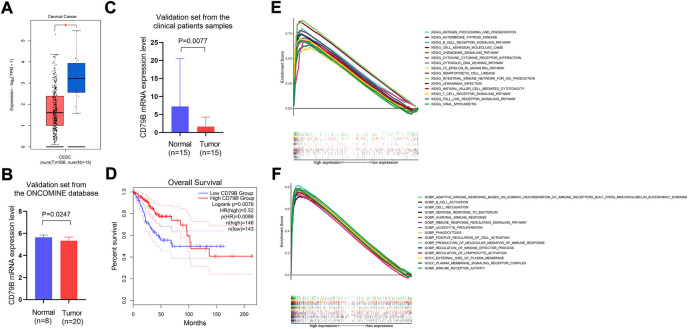
Differential expression of CD79B, survival analysis, and GSEA analysis of highly expressed CD79B. **(A)** The mRNA expression level of CD79B in CC tissues and normal tissues was analyzed using the GEPIA2 online datasets. These datasets included 13 normal samples from the TCGA and GTEx databases, respectively, as well as 306 tumor samples from the TCGA database (*p < 0.05). **(B)** mRNA expression of CD79B in CC tissues and normal tissues from the ONCOMINE database. **(C)** The mRNA expression of CD79 B was examined in CC tissues and compared to normal tissues by qRT-PCR using 30 samples from patients. An unpaired *t*-test with Welch’s correction evaluated the significance of the data. **(D)** Association between CD79B expression and overall survival in CC, based on the GEPIA2 online database. **(E and F)** GSEA analysis of the top 15 significant pathways **(E)** and enriched gene sets in c5. go.v7.4. Symbols **(F)** associated with high CD79B expression.

The analysis of the GEPIA2 data showed that the expression of CD79B mRNA in tumor tissues was significantly lower than that in normal cervical tissue (*p* < 0.05; Figure 4A). Meanwhile, the results of the validation sets from the ONCOMINE database and the qRT-PCR experiment showed that CD79 B expression was markedly lower in tumor tissues than that in normal cervical tissues (*p* = 0.0247 and *p* = 0.0077, respectively; Figures 4B,C).

Regarding the survival analysis, the results showed that the prognosis of CC patients with high CD79B expression was better than that of patients with low CD79 B expression (*p* = 0.0076; Figure 4D). The association between CD79B expression and clinicopathological features is shown in Table 1. CD79 B expression was significantly correlated with primary therapy outcome, race, histological type, the degree of differentiation (i.e., keratinizing squamous cell carcinoma present), disease-specific survival (DSS), and progression-free interval (PFI). CD79 B expression did not significantly correlate with age, tumor depth, distant metastasis, lymph node metastasis, clinical stage, histologic grade, radiation therapy, orBMI.

**TABLE 1:** Relationship between CD79B expression in cervical cancer and clinicopathological factors.

Characteristics	Total (*n* = 306)	CD79B		*p*-value
		Low (*n* = 153)	High (*n* = 153)	
**Age, median (IQR)**		46 (38, 56)	47 (39, 58)	0.450^a^
**T stage, *n* (%)**				0.404^b^
T1	140 (57.6%)	73 (30.0%)	67 (27.6%)	
T2	72 (29.6%)	29 (11.9%)	43 (17.7%)	
T3	21 (8.6%)	10 (4.1%)	11 (4.5%)	
T4	10 (4.1%)	4 (1.6%)	6 (2.5%)	
**N stage, *n* (%)**				1.000^c^
N0	134 (68.7%)	63 (32.3%)	71 (36.4%)	
N1	61 (31.3%)	29 (14.9%)	32 (16.4%)	
**M stage, *n* (%)**				0.534^b^
M0	116 (91.3%)	50 (39.4%)	66 (52.0%)	
M1	11 (8.7%)	6 (4.7%)	5 (3.9%)	
**Clinical stage, *n* (%)**				0.881^c^
Stage I	162 (54.2%)	84 (28.1%)	78 (26.1%)	
Stage II	69 (23.1%)	32 (10.7%)	37 (12.4%)	
Stage III	46 (15.4%)	22 (7.4%)	24 (8.0%)	
Stage IV	22 (7.4%)	11 (3.7%)	11 (3.7%)	
**Radiation therapy, *n* (%)**				0.726^c^
No	122 (39.9%)	63 (20.6%)	59 (19.3%)	
Yes	184 (60.1%)	90 (29.4%)	94 (30.7%)	
**Primary therapy outcome, *n* (%)**				**0.042** **^b^**
PD	23 (10.5%)	16 (7.3%)	7 (3.2%)	
SD	6 (2.7%)	2 (0.9%)	4 (1.8%)	
PR	8 (3.7%)	6 (2.7%)	2 (0.9%)	
CR	182 (83.1%)	81 (37%)	101 (46.1%)	
**Race, *n* (%)**				**0.039** ^c^
Other	51 (19.5%)	19 (7.2%)	32 (12.2%)	
White	210 (80.1%)	112 (42.9%)	98 (37.5%)	
**BMI, *n* (%)**				0.852^c^
< = 25	100 (38.5%)	52 (20.0%)	48 (18.5%)	
>25	160 (61.5%)	80 (30.8%)	80 (30.8%)	
**Histological type, *n* (%)**				**0.034** ^c^
Adenosquamous	53 (17.3%)	34 (11.1%)	19 (6.2%)	
Squamous cell carcinoma	253 (82.7%)	119 (38.9%)	134 (43.8%)	
**Histologic grade, *n* (%)**				0.579^b^
G1	19 (6.9%)	11 (4.0%)	8 (2.9%)	
G2	135 (49.3%)	69 (25.2%)	66 (24.1%)	
G3	119 (43.4%)	55 (20.1%)	64 (23.4%)	
G4	1 (0.4%)	0 (0.0%)	1 (0.4%)	
**Keratinizing squamous cell carcinoma present**				**0.047** ^c^
No	120 (39.2%)	51 (16.7%)	69 (22.5%)	
Yes	186 (60.8%)	102 (33.3%)	84 (27.5%)	
**DSS event**				**0.006** ^c^
Alive	247 (81.2%)	113 (37.4%)	134 (44.4%)	
Dead	55 (18.2%)	37 (12.3%)	18 (6.0%)	
**PFI event**				**0.043** ^c^
Alive	234 (76.5%)	109 (35.6%)	125 (40.8%)	
Dead	72 (23.5%)	44 (14.4%)	28 (9.2%)	

IQR, interquartile range; M, distant metastasis; N, lymph node metastasis; T, tumor depth; CR, complete response; PR, partial response; SD, stable disease; PD, progressive disease;BMI, body mass index; DSS, disease-specific survival; PFI, progression-free interval.

^a^ Wilcoxon rank-sum test.

^b^ Fisher’s exact test.

^c^ Chi-square test. A asf.

### 3.4 Identification of CD79B-Related functions and signaling pathways

The potential functions of the CD79 B were explored by a GSEA analysis. The results showed that the top 15 significant signaling pathways associated with high CD79 B expression were mainly immune-related pathways, such as antigen processing and presentation,B cell receptor signaling pathways, cell adhesion molecule pathways, chemokine signaling pathways, cytokine–cytokine receptor interactions, T cell receptor signaling pathways, and Toll-like receptor signaling pathways (*p* < 0.001, *q* < 0.001; Figure 4E). Highly expressed CD79 B genes were significantly enriched in 15 GO terms, such as the adaptive immune response based on somatic recombination of immune receptors and built from immunoglobulin superfamily domains; immune receptor activity;B cell activation; cell recognition; defense response against bacteria; and the humoral immune response. In total, 12 of the GO terms wereBiological Process terms, two were Cellular Component terms, and one was a Molecular Function term (*p* < 0.001, *q* < 0.001; Figure 4F).

### 3.5 Association between CD79B expression and tumor immune infiltrates

**FIGURE 5:**
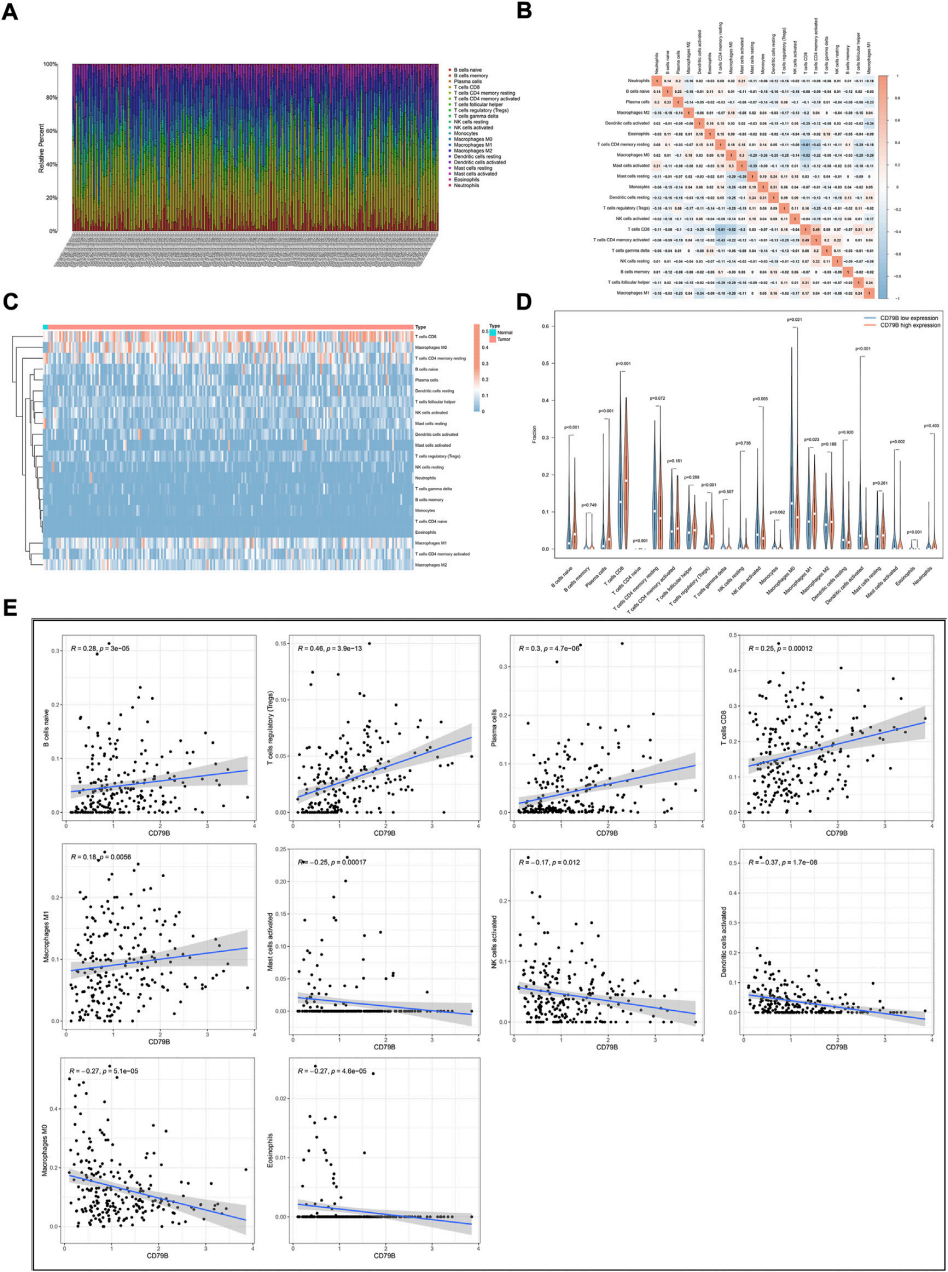
Correlation analysis of CD79B expression and TICs. **(A)** The proportions of 22 types of TICs in the TCGA-CESC cohorts. **(B)** Corrplots for the correlation among the 21 TICs populations. **(C)** Heatmaps showing the differences in TIC abundance between tumor and normal tissues in CESC cohorts. **(D)** Violin plots for the proportions of 22 TICs in samples with low and high CD79B expression. **(E)** Scatter plot showing Pearson’s correlation between TICs and CD79B expression (*p* < 0.05). The 10 types of TICs were significantly different between the high and low CD79B expression groups. The blue lines in each plot denote the best-fit linear models.

To further clarify the correlation between CD79 B expression and the tumor immune infiltrates, the CIBERSORT algorithm was applied to analyze the proportion of tumor-infiltrating immune subsets. A *p*-value for the deconvolution of each sample was counted out by Monte Carlo sampling. Removing the samples with *p* ≥ 0.05 revealed the infiltrating immune cell profiles among the CESC samples (Figures 5A,B). The difference in the infiltrating immune cells between cancerous and normal tissues in the TCGA-CESC cohorts is displayed in Figure 5C.

Compared with normal cervix tissues, CD8 T cells, M0 macrophages, M1 macrophages, and naiveB cells were increased in cancerous tissues; however, resting mast cells were reduced somewhat in most cancerous tissues (Figure 5C). The difference in the abundance of TICs between the high–CD79B expression and low–CD79 B expression groups was investigated. The results showed that 10 of the 22 TICs were significantly different between the two groups (*p* < 0.05, Figure 5D), including naiveB cells, plasma cells, CD8 T cells, regulatory T cells (Tregs), activated natural killer (NK) cells, M0 macrophages, M1 macrophages, activated dendritic cells (DCs), activated mast cells, and eosinophils. Among the TICs with different abundances, naiveB cells, plasma cells, CD8 T cells, Tregs, and M1 macrophages were positively correlated with CD79B expression, whereas activated NK cells, M0 macrophages, activated dendritic cells, activated mast cells, and eosinophils were negatively correlated with CD79B expression (Figure 5E).

**FIGURE 6:**
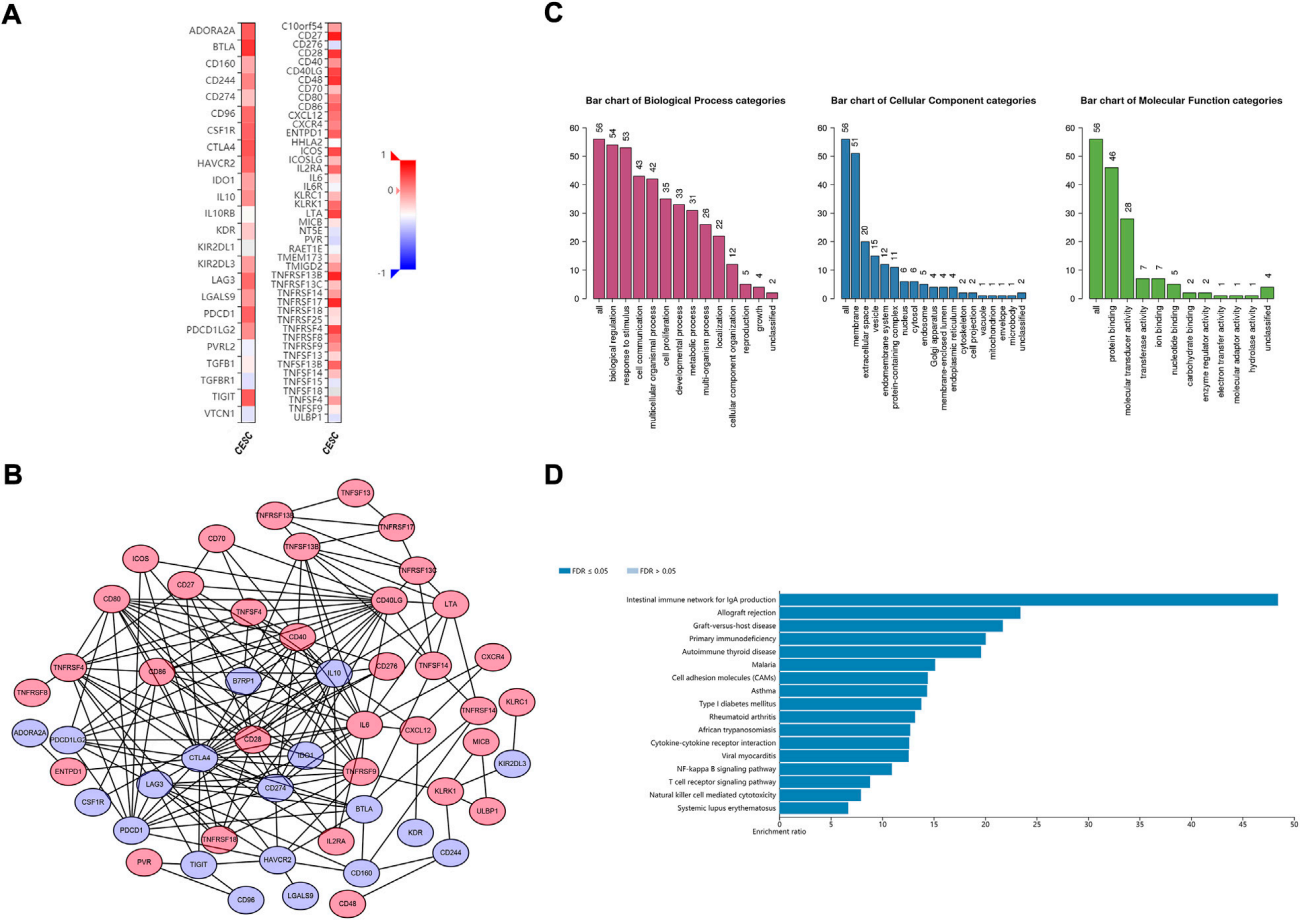
Identification and analysis of the CD79B-associated immunomodulators. **(A)** Heatmaps of CD79B-associated immunoinhibitors (left panel) and immunostimulators (right panel) in the CESC cohorts. **(B)** The PPI network concerning the 52 CD79B-associated immunomodulators, including immunoinhibitors (blue node) and immunostimulators (red node), as visualized by Cytoscape **(C and D)** GO **(C)** and KEGG pathway **(D)** enrichment analysis results for 52 immunomodulators from the PPI network based on the CESC cohorts that are highly likely to have an interactive relationship.

The potential CD79B-associated immunomodulators (including immunoinhibitors and immunostimulators) in the CESC data were investigated in hopes of producing insights into the relationship between CD79B and immune infiltration and regulation. A total of 38 immunostimulators (C10orf54,CD27, CD28, CD40, CD40LG, CD48,CD70, CD80, CD86, CD276, CXCL12, CXCR4, ENTPD1, ICOS, ICOSLG, IL2RA, IL6, KLRC1, KLRK1, LTA, MICB, PVR, TMEM173, TMIGD2, TNFRSF4, TNFRSF8, TNFRSF9, TNFRSF13B, TNFRSF13C, TNFRSF14, TNFRSF17, TNFRSF18, TNFRSF25, TNFSF4, TNFSF13, TNFSF13B, TNFSF14, and ULBP1) and 19 immunoinhibitors (ADORA2A,BTLA, CD96, CD160, CD244, CD274, CSF1R, CTLA4, HAVCR2, IDO1, IL10, KDR, KIR2DL3, LAG3, LGALS9, PDCD1, PDCD1LG2, TGFBR1, and TIGIT) that were significantly related to CD79B expression in the CESC data were identified (*p* < 0.05, Figure 6A). Among these 57 CD79B-associated immunomodulators, 52 were deemed highly likely to be involved in an interactive relationship (as supported by a strong confidence value), and these were used to build the PPI network (Figure 6B). Furthermore, GO enrichment analyses of these 52 genes demonstrated that their functions were mainly involved in biological regulation and stimulus response (Figure 6C). The results of the KEGG pathway analysis showed that the NF-kappaB signaling pathway, T cell receptor signaling pathway, and natural killer cell-mediated cytotoxicity were correlated with CD79 B-mediated immune events (Figure 6D).

Overall, the results described above indicate that CD79B widely participates in modulating tumor immune cells and affects the immune activity in the tumor microenvironment of cervical cancer.

### 3.6 Establishment and validation of gene prognostic signature

**FIGURE 7:**
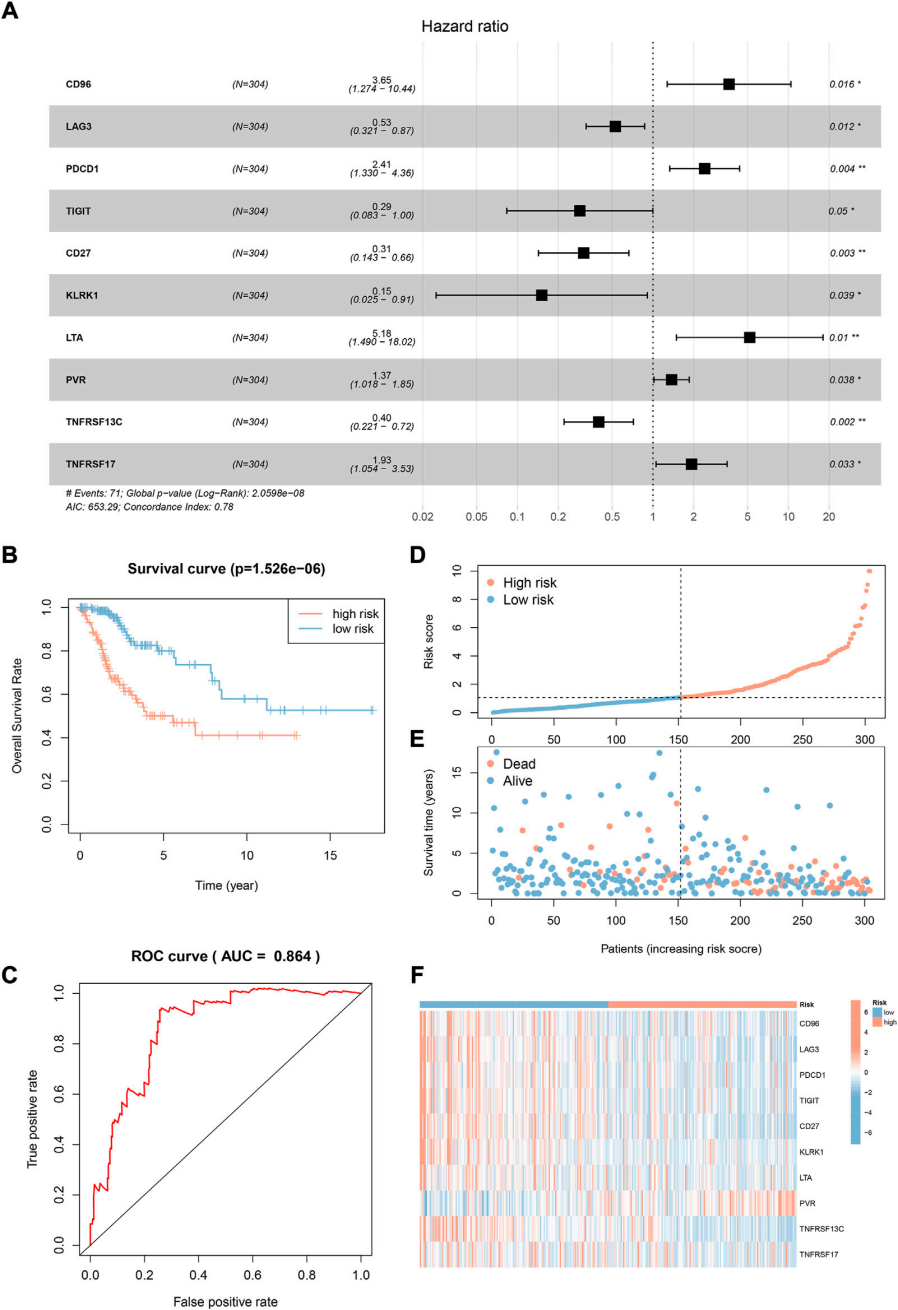
Construction and validation of a prognostic signature based on CD79B-associated immunomodulators. **(A)** The hazard ratios of the 10 genes used to establish the prognostic signature. **(B)** Kaplan-Meier curve between the high-risk-score and low-risk-score groups from the CESC cohorts (*p* = 1.526e-06). **(C)** ROC curve describing the predictive accuracy of the prognostic signature in the CESC cohorts. **(D)** Distribution of risk scores in the CESC cohorts. **(E)** Survival status of CESC patients in the low-risk and high-risk groups. **(F)** Expression profile of 10 CD79B-associated immunomodulatory genes from the CESC cohorts.

In the TCGA CESC dataset, 24 CD79 B-associated immunomodulators were found to correlate with the OS of CC patients by univariate Cox proportion hazard regression analysis (*p* < 0.05, Supplementary Figure S1). Meanwhile, the Akaike information criterion (AIC) was applied to screen important prognostic immune genes from the CD79 B-associated immunomodulators in the multivariate Cox proportion hazard regression analysis. We selected 10 genes (CD96, LAG3, PDCD1, TIGIT, CD27, KLRK1, LTA, PVR, TNFRSF13C, and TNFRSF17) (log-rank test, *p* = 2.0598e-08) (Figure 7A) and established a 10-gene optimal prognostic signature to investigate the prognostic values of CD79B-associated immunomodulators in CC. The biological functions and the risk coefficients of the 10 genes are shown in Table 2. We obtained the risk scores of the immune gene signature in each CESC patient according to the proposed formula (Dai et al., 2021) and divided the patients into the high-risk and low-risk groups. The survival time of patients with low-risk scores was significantly longer than those with high-risk scores, which confirmed the prognostic value of the risk score **(** *p* = 1.526e−06, Figure 7B).

**TABLE 2:** Function and risk coefficients of the genes involved in the prognostic signature.

Gene symbol	Full name	Function	Risk coefficient
CD96	CD96	Adhesive interactions of activated T and NK cells 1.29380 during the late phase of the immune response	
LAG3	Lymphocyte activating 3	Inhibitory receptor on antigen-activated T-cells	–0.63862
PDCD1	Programmed cell death 1	Inhibitory receptor on antigen activated T-cells to induction and maintenance of immune tolerance to self	0.87924
TIGIT	T cell immunoreceptor with and Ig and ITIM domains	Binds to PVR, decreases the secretion of IL12B, suppresses T-cell activation	–1.24275
CD27	CD27	Receptor for CD70/CD27L to promote the survival of activated T cells and induce apoptosis	–1.17728
KLRK1	Killer cell lectin like receptor K1	Activating and costimulatory receptor in immunosurveillance	–1.89030
LTA	Lymphotoxin alpha	Cytokine binding to TNFRSF1A/TNFR1, TNFRSF1B/TNFBR, and TNFRSF14/HVEM	1.64513
PVR	Poliovirus receptor	Cell adhesion and regulation of immune response	0.31694
TNFRSF13C	TNF receptor superfamily member 13C	B-cell receptor specific for TNFSF13B/TALL1/BAFF/BLyS to promote theB-cell response	–0.92140
TNFRSF17	TNF receptor superfamily member 17	PromotesB cell survival and plays a role in the regulation of humoral immunity	0.65649

We also calculated the area under the ROC curve (AUC) value of the risk score to assess its predictive sensitivity and specificity in the prognosis of CC patients (AUC = 0.864, Figure 7C). These results demonstrated that the prognostic risk model based on the 10 CD79B-related immunomodulators was considered reliable. The distribution of the risk scores, the survival status of the patients, and the 10-gene expression profiles were also acquired and these results showed that the occurrence of mortality depended on the risk score (Figures 7D–F). In addition, CD96, LAG3, PDCD1, TIGIT, CD27, KLRK1, LTA, TNFRSF13C, and TNFRSF17 were highly expressed in the low-risk group, and PVR was up-regulated in the high-risk group (Figure 7F).

**FIGURE 8:**
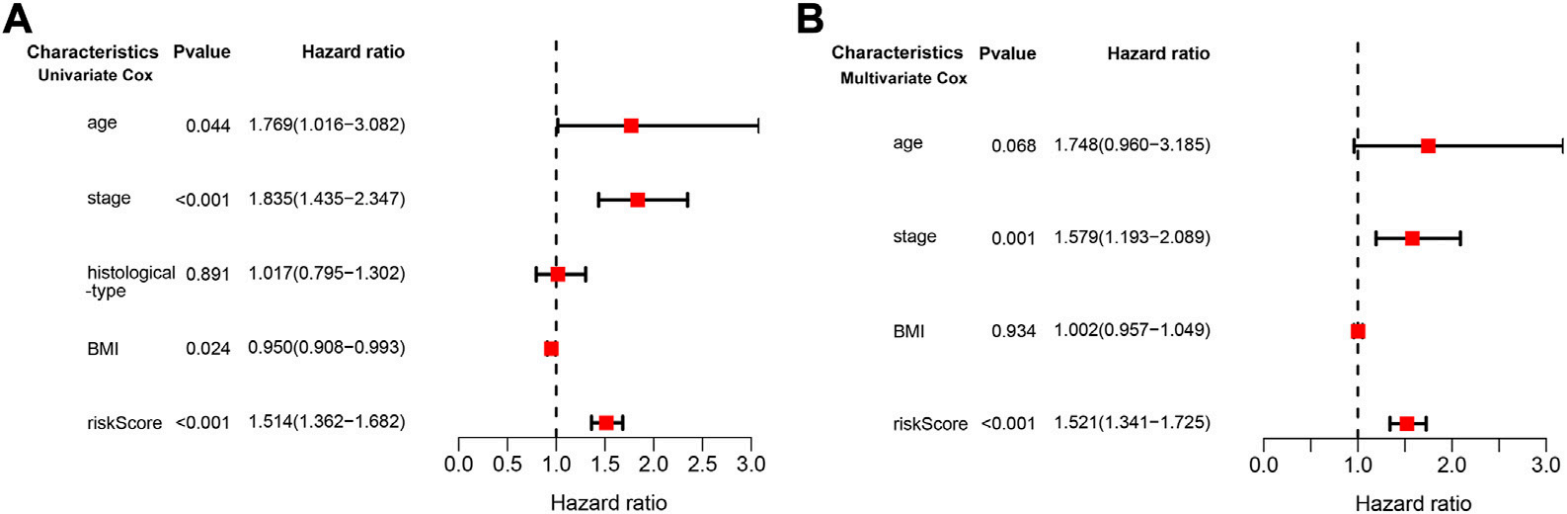
Cox regression analysis of the association between clinical factors and OS in the CESC cohorts. **(A)** The forest map from the univariate Cox regression analysis. **(B)** The forest map from the multivariate Cox regression analysis.

We conducted univariate and multivariate Cox regression analyses to assess whether the risk model of the above 10 CD79B-related immunomodulators is an independent prognostic factor for CC. In the univariate model, the hazard ratio (HR) of the risk score was 1.514 and the 95% confidence interval (CI) was 1.362–1.682 (*p* < 0.001; Figure 8A), indicating that risk score, age, stage, andBMI were significantly associated with the survival of patients with CC. In the multivariate model, the HR of the risk score was 1.521, and the 95% CI was 1.341–1.725 (*p* < 0.001), indicating that the risk score and stage were significant independent prognostic predictors (Figure 8B).

### 3.7 Construction and evaluation of a prognostic nomogram

**FIGURE 9:**
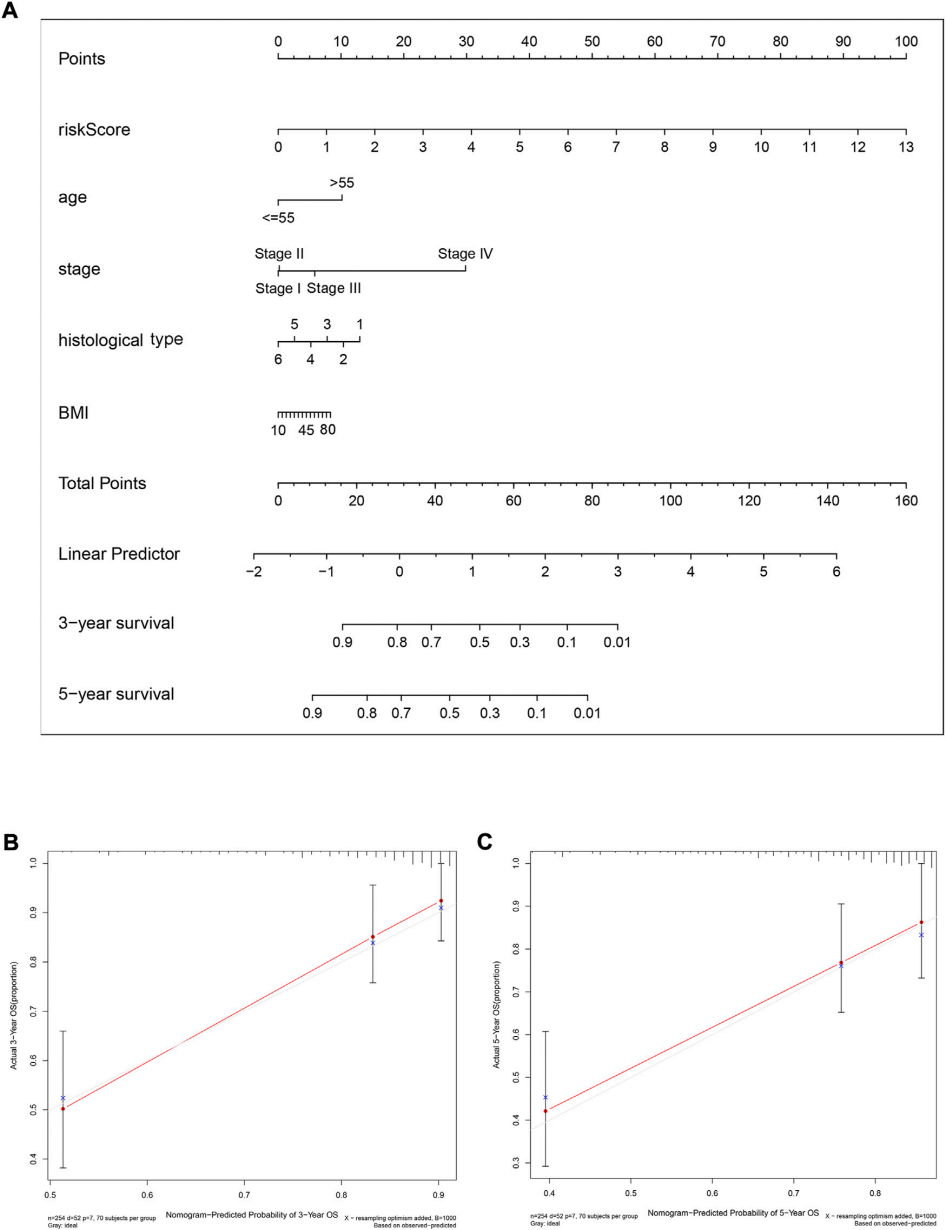
A nomogram (inclusive of risk score) for predicting OS in cervical cancer patients. **(A)** A nomogram comprising the risk score and other clinical factors for predicting the 3-year and 5-year OS of CC patients. **(B and C)** Calibration plots of the nomogram for predicting 3-year **(B)** and 5-year **(C)** survival of CC patients. The *x*-axis represents nomogram-predicted survival, and the *y*-axis represents actual survival. The C-index of the nomogram for predicting survival is 0.83.

To predict the 3-year and 5-year OS probability of CC patients, a prognostic nomogram, including the features of risk score, age, stage, histological type, andBMI, was established using the multivariate Cox regression analysis (Figure 9A). The concordance index (C-index), i.e., the indicator for evaluating the predictive discrimination of the prognostic nomogram, was 0.83. The calibration curves showed acceptable accuracy: the nomogram-predicted probability (solid line) closely matched the actual reference line (dashed line) for the 3-year and 5-year survival (Figures 9B,C). Taken together, these results indicate that the risk model nomogram was effective for predicting the OS in CC patients.

## 4 Discussion

Immunotherapy is one of the best strategies for cancer treatment (Riley et al., 2019). In addition to the factors intrinsic to the tumor itself, the tumor microenvironment can influence the efficacy of immunotherapy (Wang et al., 2018). Thus, the use of immunotherapeutic strategies to target the tumor microenvironment has attracted more and more interest (Yang et al., 2021). In this study, we explored prognosis-related genes in the TME that contributed to overall survival in cervical cancer patients. Ten differential genes (CD79A, CD79B, CCR2, CD3D, CD3E, CXCL9, CXCR3,BTK, CD28, CCL5) were identified as associated with patient survival, but further analysis revealed that all but one—CD79B—were not significantly associated with the prognosis of CC (data not shown). CCR2(Santos et al., 2016), CD28 (Escarra-Senmarti et al., 2017), and CXCR3 (Chen et al., 2021) were shown to play an important immunological role in CC in other studies. Therefore, we selected CD79B for the subsequent series of bioinformatics analyses and investigated whether CD79B might be a prognostic and therapeutic biomarker in CC patients.

CD79B, a transmembrane heterodimer, is a part of theB-cell antigen receptor (BCR), which is key to the successful development and maintenance of matureB cells (Chu and Arber, 2001; Ormhoj et al., 2019). It has also been reported that CD79B was differentially expressed in tumor tissues; for example, CD79B expression was found to be low inB cell chronic lymphocytic leukemia (B-CLL) (Contri et al., 2005). However, it maintains high expression in most subtypes of non-Hodgkin lymphoma, such as mantle cell lymphoma, diffuse largeB-cell lymphoma, andBurkitt’s lymphoma (Ormhoj et al., 2019). In our study, based on the results of public database analyses and clinical specimen validation experiments, we found that CD79B expression was lower in CC tissues than in normal cervical tissues, which means that higher expression of CD79B may play a potential role in controlling the development of CC. The analysis of CD79B expression and clinicopathological factors demonstrated that CD79B expression was related to primary therapy outcome, race, histological type, the degree of differentiation, disease-specific survival, and progression-free interval, whereas there was no statistically significant correlation between CD79B expression and age, tumor depth, distant metastasis, lymph node metastasis, clinical stage, histologic grade, radiation therapy, orBMI. These results suggest that detecting CD79B expression may be significant for differentiating cervical cancer types and for predicting patient outcomes and prognosis, but not for indicating the extent of metastasis, progression, or infiltration. Furthermore, the survival analysis revealed that CD79B was a protective factor for patients with CC. This is the first study to report that a high level of CD79 B expression is related to better prognosis, which indicates that high CD79B expression is necessary for efficacious anti-tumor responses.

Previous studies have reported thatB-cell receptor (BCR) activation was essential forB cells’ differentiation, activation, and function (Tsui et al., 2018; Jing et al., 2020). CD79B is one of the components of theBCR signaling complex, and theBCR signaling pathway is dependent on CD79B activity (Nayyar et al., 2019). In this study, we found that a high level of CD79B expression was significantly associated with immune-related pathways, especially theB-cell receptor, T-cell receptor, and Toll-like receptor signaling pathways. We also observed that the functions of high CD79B expression were primarily involved in the adaptive immune response,B cell activation, cell recognition, and immune receptor activity. Thus, the above findings strongly suggest that CD79B-mediated immune-related activities participate in controlling CC.

The tumor microenvironment may facilitate immunosuppression and lead to the immune escape of cancer cells (Nakamura and Smyth, 2020). Moreover, tumor-infiltrating immune cells, as a component of the TME, are associated with tumor progress, prognosis, and response to immunotherapy in many cancers (Lei et al., 2020; Zhang and Zhang, 2020). Cytotoxic CD8^+^ T cells can kill tumor cells and control tumor growth. In most cancer types, CD8^+^ T cell infiltration in tumors predicts a good prognosis (Disis et al., 2019; Shimizu et al., 2019), while regulatory T (Treg) cells are known to suppress the anti-tumor immune responses and are usually correlated with a poor outcome (Sharma et al., 2019). The role ofB lymphocytes in mediating the anti-tumor immune response (cytokine production, antibody production from plasma cells, and induction of T cell activation, as well as proliferation *via* antigen presentation) has been widely demonstrated (Chen et al., 2020). NaiveB cells can differentiate into plasma cells with a stronger antibody-secreting ability, which is the basis for generating humoral immunity (Akkaya et al., 2020). In addition, tumor-induced regulatoryB (Breg) cells, which produce pro-inflammatory factors and promote Treg-cell differentiation, can promote immune suppression and support tumor progression (Matsushita, 2019).

A recent study showed that infiltratingB cells in tumors can be considered a predictor of patient survival (Wouters and Nelson, 2018). In breast cancer,B cells play a role in negatively regulating immune responses and promoting tumor evasion by PD-L1, which is associated with poor prognosis (Guan et al., 2016). However, in other solid cancers (ovarian cancer, colorectal cancer, and some types of non–small-cell lung cancer), tumor-infiltratingB cells are essential for good prognosis (Flynn et al., 2017). We found that CD79B expression was positively correlated with the infiltration level of naiveB cells, plasma cells, and CD8^+^ T cells. This finding may explain why CD79B plays a protective role in CC. We also found that CD79B expression was positively correlated with Treg infiltration levels, which may indicate that they can suppress the anti-tumor immune response. Although the number of infiltrating cytotoxic CD8^+^ T cells is increased in CC samples with high levels of CD79B expression, their anti-tumor function may be limited to some degree due to the increased Treg infiltration.

Macrophages can stimulate the proliferation and differentiation of naive CD8^+^ T cells into memory T cells, and tumor-infiltrating M1 macrophages, the anti-cancer phenotype of macrophages, play an antitumoral role (Gardner and Ruffell, 2016; Vitale et al., 2019). We showed that CD79B expression was positively correlated with the infiltration level of M1 macrophages and negatively correlated with M0 macrophages, suggesting that CD79B may have an influence on the polarization of macrophages. A

NK cells play a critical role in anti-tumor immunotherapy by directly killing tumor cells (Watkins-Schulz et al., 2019). The infiltration density of NK cells in tumors such as hepatocellular carcinoma, renal cell carcinoma, and breast cancer has been correlated with better clinical outcomes (Verma et al., 2015; Kremer et al., 2017; Park et al., 2020). Dendritic cells are antigen-presenting cells and can regulate cell-mediated immune responses (Gardner and Ruffell, 2016). Several studies have reported that mature tumor-infiltrating dendritic cells are also associated with a favorable prognosis in various cancers (Laoui et al., 2016). Our results showed that CD79B expression was negatively correlated with activated NK cells and activated dendritic cells. This represents a contrast to other recent findings. In future studies, we will continue to focus on and verify the relationship between CD79B expression and NK cells, as well as dendritic cells. Our study also revealed that CD79 B expression was significantly associated with tumor-infiltrating immunomodulators (19 immunoinhibitors and 38 immunostimulators) in CC. Taken together, we could speculate from these results that CD79B may affect or regulate the immune cells in the TME of CC patients. Therefore, CD79B might be a potential immunotherapeutic target for CC.

Immune-related gene signatures have been shown to have prognostic value for clinical outcomes in various cancer types (Catacchio et al., 2018). Nomograms can generate individual probabilities of clinical events by integrating different prognostic and deterministic variables to facilitate personalized medicine and aid clinical decisions (Balachandran et al., 2015), and they are therefore widely used as prognostic tools in oncology. CD79B, as we have seen, is also associated with immune response activities. Through a series of bioinformatics analyses, we found that CD79B might be a prognostic and therapeutic biomarker in CC patients. We therefore established a ten-gene immune prognostic signature from CD79B-related immunomodulators. The prognostic signature had a high degree of accuracy when measured against the TCGA-CESC dataset, as confirmed by an AUC of 0.864 in the ROC curve, and the risk scores derived from the signature were significantly correlated with survival in CC patients. Finally, with the addition of clinical features, we constructed a nomogram with a C-index of 0.83. This nomogram may provide clinicians with a convenient and accurate method for assessing the individualized prognosis of CC patients.

To our knowledge, this is the first time a 10-gene prognostic signature based on CD79B-related immunomodulators has been established, and the first time that the resulting nomogram has been applied to obtain personalized prognoses for CC patients. However, this study has some limitations: First, it is not enough to verify the differential expression of CD79B in cervical tissue by RT-qPCR experiments; its core mechanism should be investigated with follow-up experiments. Second, although our results have shown good predictive potentiality and clinical value of the 10-gene prognostic signature, prospective studies will be needed to prove the clinical application and prognostic value of this model in patients with CC.

In conclusion, our findings suggested that CD79B expression was down-regulated in CC tissues compared to normal cervical tissues, and that high CD79B expression in CC patients predicts a good prognosis. In CC tissues, CD79B expression was associated with infiltration of multiple immune cells, such asB cells, T cells, and macrophages, suggesting it may play a role in regulating the tumor immune microenvironment. The 10-gene prognostic signature based on CD79B-associated immunomodulators independently predicted overall survival. In the future, with prospective validation, the 10-gene immune signature may improve predictive accuracy and guide individualized treatment and medical decisions for CC patients. Furthermore, the prognostic genes associated with the immune activity ofB cells should be identified; this may be a new research direction for CC.

## Data Availability

The original contributions presented in the study are included in the article/Supplementary Materials, further inquiries can be directed to the corresponding authors.
