# Supplementary material for: A novel ten-gene prognostic signature for cervical cancer based on CD79B-related immunomodulators
Source: Front Genet. 2022 Nov 2;13:933798. doi: 10.3389/fgene.2022.933798 (PMC9666757; doi:10.3389/fgene.2022.933798)
Supplement: Supplementary file 1 [file Table3.doc]

**Supplementary Table 3 | Multivariate cox regression analysis showing associations between 122 DEGs and OS in cervical cancer.**

| **Gene** | **Multivariate cox regression analysis** | | | | |
| --- | --- | --- | --- | --- | --- |
|  | **Coef** | **HR** | **HR.95L** | **HR.95H** | ***P*-value** |
| BTNL8 | -5.36307031 | 0.004686495 | 0.002793103 | 0.007863381 | 1.03E-91 |
| LY9 | -4.913898583 | 0.007343802 | 0.004367904 | 0.012347211 | 1.05E-76 |
| CD6 | -5.332880846 | 0.004830135 | 0.002836327 | 0.008225498 | 7.98E-86 |
| CD27 | 6.178751302 | 482.3892215 | 289.8495979 | 802.827959 | 6.23E-125 |
| CD1E | -6.343896108 | 0.001757442 | 0.001047861 | 0.00294753 | 9.44E-128 |
| JAK3 | 2.833794313 | 17.00987934 | 10.0027157 | 28.92574417 | 1.30E-25 |
| GRAP2 | -16.30105259 | 8.33E-08 | 4.91E-08 | 1.41E-07 | 0 |
| SLAMF6 | -0.963649053 | 0.381498236 | 0.224979292 | 0.646908001 | 0.000348287 |
| SELPLG | -3.788608077 | 0.022627075 | 0.013436256 | 0.038104702 | 4.66E-46 |
| ZNF831 | -3.203316789 | 0.040627228 | 0.02359068 | 0.069967108 | 7.39E-31 |
| CD5 | 10.26607394 | 28740.82724 | 16910.35593 | 48847.8867 | 6.48771511948662e-315 |
| CD79A | -5.963569924 | 0.002570718 | 0.00157247 | 0.004202682 | 5.48E-125 |
| BIN2 | -2.552601671 | 0.077878787 | 0.04642016 | 0.130656713 | 4.08E-22 |
| LILRA4 | -2.119746401 | 0.120062072 | 0.07180979 | 0.20073727 | 6.31E-16 |
| FOXP3 | -4.145910198 | 0.015829022 | 0.009383882 | 0.026700882 | 1.82E-54 |
| CD1C | 3.181701432 | 24.0877023 | 14.49477757 | 40.02941054 | 1.19E-34 |
| SELL | -5.631676818 | 0.003582563 | 0.002185443 | 0.00587284 | 1.80E-110 |
| TIGIT | -11.35444401 | 1.17E-05 | 6.96E-06 | 1.97E-05 | 0 |
| CD79B | -3.889192441 | 0.020461864 | 0.012466752 | 0.033584359 | 2.10E-53 |
| CCR2 | -2.690481575 | 0.067848258 | 0.040748866 | 0.112969671 | 4.51E-25 |
| CHIT1 | -1.030546299 | 0.356811981 | 0.214531719 | 0.593454388 | 7.18E-05 |
| SLAMF1 | 2.499956678 | 12.1819662 | 7.27940727 | 20.38631649 | 1.80E-21 |
| POU2AF1 | -3.290895765 | 0.037220494 | 0.022738389 | 0.060926266 | 3.84E-39 |
| CD1B | -3.329490886 | 0.035811332 | 0.02149625 | 0.059659314 | 1.97E-37 |
| GNG8 | -1.624235169 | 0.197062337 | 0.119222286 | 0.325724039 | 2.38E-10 |
| SIT1 | -4.352703922 | 0.012871961 | 0.007671011 | 0.021599157 | 4.94E-61 |
| P2RY8 | 3.6161085 | 37.19255101 | 22.67641043 | 61.0010943 | 1.54E-46 |
| PSTPIP1 | -5.221458748 | 0.005399447 | 0.003237403 | 0.009005374 | 4.83E-89 |
| FCMR | 3.093700958 | 22.05856492 | 13.41218673 | 36.27896749 | 3.64E-34 |
| PPP1R16B | -1.969780075 | 0.13948753 | 0.084928365 | 0.229096262 | 7.19E-15 |
| P2RY13 | -3.587582877 | 0.02766512 | 0.016541445 | 0.046269164 | 1.49E-42 |
| PYHIN1 | -3.945523123 | 0.019341096 | 0.011475179 | 0.032598879 | 1.21E-49 |
| PTGDS | 1.592065245 | 4.913886838 | 2.986753517 | 8.084458164 | 3.67E-10 |
| PLD4 | 0.858796235 | 2.360317715 | 1.420885915 | 3.920863496 | 0.000911308 |
| P2RY10 | -2.022292055 | 0.13235176 | 0.079496406 | 0.220349437 | 7.51E-15 |
| CD300LF | -3.425904686 | 0.032519847 | 0.019570252 | 0.054038164 | 6.55E-40 |
| RHOH | -4.476729801 | 0.011370536 | 0.006871845 | 0.018814321 | 5.50E-68 |
| CD3D | 5.146708706 | 171.8649004 | 101.3825044 | 291.3475472 | 2.01E-81 |
| RIPOR2 | -2.140296582 | 0.117619954 | 0.07087024 | 0.195208223 | 1.23E-16 |
| KLRD1 | -3.416945314 | 0.032812514 | 0.019624118 | 0.054864177 | 8.47E-39 |
| SCUBE1 | -2.743668187 | 0.064333925 | 0.038934013 | 0.106304323 | 9.39E-27 |
| PLA2G7 | -3.246911275 | 0.038894156 | 0.02319115 | 0.065229855 | 8.26E-35 |
| SCML4 | 1.537529817 | 4.653082101 | 2.808716249 | 7.708565452 | 2.38E-09 |
| GZMM | -1.14738153 | 0.31746696 | 0.189701459 | 0.531283581 | 1.26E-05 |
| AMPD1 | -5.89961591 | 0.002740497 | 0.001683497 | 0.004461146 | 1.75E-124 |
| MS4A6A | 2.489820814 | 12.0591151 | 7.231460003 | 20.10966761 | 1.39E-21 |
| CD2 | 1.875512058 | 6.524159016 | 3.853895222 | 11.04457916 | 2.89E-12 |
| ABCD2 | -1.241035233 | 0.289084793 | 0.176705915 | 0.472932769 | 7.75E-07 |
| IL12RB1 | -3.636817158 | 0.026336034 | 0.015745496 | 0.044049846 | 1.15E-43 |
| BLK | 4.62850528 | 102.3609488 | 61.61076653 | 170.0638448 | 2.04E-71 |
| ASGR2 | -1.793627404 | 0.166355635 | 0.101560606 | 0.272489485 | 1.05E-12 |
| SLA2 | -4.214515524 | 0.01477948 | 0.008844031 | 0.024698357 | 3.18E-58 |
| HLA-DQA1 | 3.60654672 | 36.83861885 | 21.90449616 | 61.95457905 | 4.15E-42 |
| JCHAIN | 2.175611318 | 8.807567699 | 5.36749536 | 14.45241096 | 7.30E-18 |
| GPR171 | -4.867108679 | 0.007695584 | 0.00458509 | 0.012916214 | 8.82E-76 |
| TTC24 | 1.420332447 | 4.138496044 | 2.471563059 | 6.929683401 | 6.65E-08 |
| MPEG1 | 3.813795723 | 45.32214311 | 27.40414253 | 74.95569889 | 6.21E-50 |
| CYSLTR2 | -1.489322626 | 0.225525369 | 0.137179371 | 0.370767788 | 4.32E-09 |
| TCL1A | 3.39928113 | 29.94256748 | 18.09317482 | 49.55224036 | 6.22E-40 |
| PILRA | -3.42420824 | 0.032575062 | 0.019607272 | 0.054119446 | 6.68E-40 |
| P2RX1 | -1.975711841 | 0.138662571 | 0.084747257 | 0.226878241 | 3.70E-15 |
| CYTIP | -1.52070427 | 0.218557909 | 0.129409771 | 0.369118647 | 1.29E-08 |
| LST1 | -6.777744172 | 0.001138841 | 0.000678039 | 0.001912809 | 9.80E-145 |
| TESPA1 | -3.120457543 | 0.044136969 | 0.02634295 | 0.073950415 | 2.14E-32 |
| RASGRP2 | -1.494176317 | 0.224433391 | 0.136649229 | 0.368610546 | 3.58E-09 |
| IGSF6 | -6.550636948 | 0.001429205 | 0.000865087 | 0.002361182 | 3.01E-144 |
| TNFSF8 | 2.775256882 | 16.04274757 | 9.755151917 | 26.38295658 | 7.90E-28 |
| TREM2 | -3.638161808 | 0.026300645 | 0.015909074 | 0.043479835 | 1.14E-45 |
| VCAM1 | -1.439918029 | 0.236947181 | 0.145489585 | 0.385896807 | 7.19E-09 |
| CD3E | 6.989399047 | 1085.069204 | 651.8054398 | 1806.329168 | 3.86E-159 |
| CXCL9 | -4.541464746 | 0.010657784 | 0.006367071 | 0.01783997 | 6.81E-67 |
| PTPN7 | 10.48674946 | 35837.47675 | 21504.7413 | 59722.86399 | 0 |
| IL16 | 3.178609436 | 24.01333823 | 14.39627039 | 40.05484735 | 4.15E-34 |
| RASSF4 | 5.060134552 | 157.6117219 | 94.89273258 | 261.7845878 | 4.41E-85 |
| LAPTM5 | -3.345827951 | 0.035231034 | 0.021135193 | 0.05872791 | 1.07E-37 |
| SELP | 1.291743989 | 3.639127624 | 2.218989357 | 5.968144835 | 3.09E-07 |
| SNX20 | -9.040656841 | 0.000118493 | 7.06E-05 | 0.000198895 | 1.59E-256 |
| C20orf141 | 3.637005203 | 37.97793006 | 23.01518463 | 62.66832942 | 5.75E-46 |
| KLHL6 | -1.026768144 | 0.358162622 | 0.216452134 | 0.592650493 | 6.44E-05 |
| ITK | 2.208469256 | 9.101773243 | 5.456100721 | 15.18342135 | 2.71E-17 |
| TMEM273 | 3.082510125 | 21.8130873 | 13.24848025 | 35.91436668 | 8.64E-34 |
| CD1A | 3.391350024 | 29.70602908 | 17.98200869 | 49.07394822 | 5.06E-40 |
| CXCR3 | 5.864661257 | 352.3627756 | 211.4027695 | 587.312673 | 4.27E-112 |
| CLNK | 2.843389655 | 17.17388051 | 10.36731474 | 28.44923486 | 2.41E-28 |
| CD8A | 4.89682834 | 133.8645329 | 79.09044847 | 226.5724056 | 2.57E-74 |
| JAML | 2.960863918 | 19.31465083 | 11.69509057 | 31.8984906 | 6.04E-31 |
| IRF4 | 6.27246727 | 529.7828842 | 322.7316618 | 869.6695664 | 8.20E-136 |
| KCNA3 | 6.240663623 | 513.1989684 | 309.7994422 | 850.1409145 | 9.92E-130 |
| JAKMIP1 | -1.042443214 | 0.352592171 | 0.21120717 | 0.588622245 | 6.70E-05 |
| NFAM1 | 3.42898703 | 30.84538149 | 18.91411448 | 50.30304538 | 5.72E-43 |
| CLEC9A | -5.170332294 | 0.00568268 | 0.003427053 | 0.009422922 | 2.57E-89 |
| TLR10 | 8.597066193 | 5415.747501 | 3338.979505 | 8784.217138 | 6.16E-266 |
| RAB33A | 3.213964867 | 24.87752703 | 15.09989669 | 40.98646259 | 1.71E-36 |
| LAX1 | 2.623453323 | 13.78323947 | 8.461856687 | 22.45106452 | 5.70E-26 |
| NCF1 | 2.202579568 | 9.048324187 | 5.446581596 | 15.03184505 | 1.82E-17 |
| BTK | -6.347907813 | 0.001750405 | 0.00104401 | 0.002934762 | 4.52E-128 |
| CXorf65 | 1.181982507 | 3.260832424 | 1.954282384 | 5.440886222 | 6.04E-06 |
| CD28 | 5.965727801 | 389.8366484 | 237.2431068 | 640.5775681 | 1.47E-122 |
| TIMD4 | -2.471676741 | 0.084443151 | 0.050894661 | 0.140105968 | 1.09E-21 |
| THEMIS | 4.53863279 | 93.56279264 | 55.99552796 | 156.3338446 | 2.89E-67 |
| IGLL5 | 5.667634715 | 289.3493307 | 178.4499106 | 469.1682664 | 6.88E-117 |
| CASS4 | 5.772113262 | 321.2158291 | 193.7432713 | 532.5584117 | 6.66E-111 |
| ADA2 | 8.31759238 | 4095.288231 | 2474.934295 | 6776.497352 | 7.31E-230 |
| CSF2RB | -1.89808969 | 0.149854615 | 0.091465737 | 0.245517132 | 4.87E-14 |
| SIGLEC1 | 1.315459104 | 3.726461425 | 2.255463022 | 6.156835477 | 2.82E-07 |
| SIGLEC10 | 1.64447426 | 5.178286759 | 3.11052436 | 8.620621687 | 2.55E-10 |
| CCL5 | 6.393312461 | 597.8336039 | 358.1104622 | 998.030093 | 4.86E-132 |
| CLECL1 | 1.339722207 | 3.817982751 | 2.306987043 | 6.318627724 | 1.87E-07 |
| TBX21 | -1.219838348 | 0.295277895 | 0.177887846 | 0.490134866 | 2.38E-06 |
| GMFG | 12.36238602 | 233838.5088 | 141509.4243 | 386408.5272 | 0 |
| SH2D1A | -2.608326355 | 0.073657718 | 0.043508916 | 0.124697644 | 2.72E-22 |
| FERMT3 | 6.459248339 | 638.5808795 | 383.3841567 | 1063.647343 | 6.48E-136 |
| CD72 | -1.818184535 | 0.16232017 | 0.097815249 | 0.269363294 | 1.98E-12 |
| CD37 | -2.686743042 | 0.068102385 | 0.041194941 | 0.112585059 | 1.12E-25 |
| CLEC10A | 1.962307379 | 7.115726813 | 4.313120078 | 11.73942927 | 1.56E-14 |
| TRAT1 | 5.555256764 | 258.5933534 | 153.8379723 | 434.6815122 | 1.38E-97 |
| GZMK | -1.039639142 | 0.353582252 | 0.212047442 | 0.589586971 | 6.74E-05 |
| CTLA4 | 3.809967749 | 45.14898276 | 27.28152979 | 74.71834093 | 1.03E-49 |
| ARHGAP9 | -2.863808389 | 0.057051073 | 0.034305713 | 0.094877055 | 2.58E-28 |
| AIF1 | 3.652649523 | 38.57674073 | 23.03966723 | 64.59142447 | 7.34E-44 |
| TNFRSF8 | 2.9526052 | 19.15579345 | 11.77564528 | 31.16130065 | 1.28E-32 |
| CD40LG | -1.14493966 | 0.31824312 | 0.1917386 | 0.528212282 | 9.47E-06 |

Abbreviations:DGEs,differentially expressed genes; OS, overall survival; Coef ,β coefficient; HR,hazard ratio.
