# Supplementary figures and images for: A novel ten-gene prognostic signature for cervical cancer based on CD79B-related immunomodulators
Source: Front Genet. 2022 Nov 2;13:933798. doi: 10.3389/fgene.2022.933798 (PMC9666757; doi:10.3389/fgene.2022.933798)

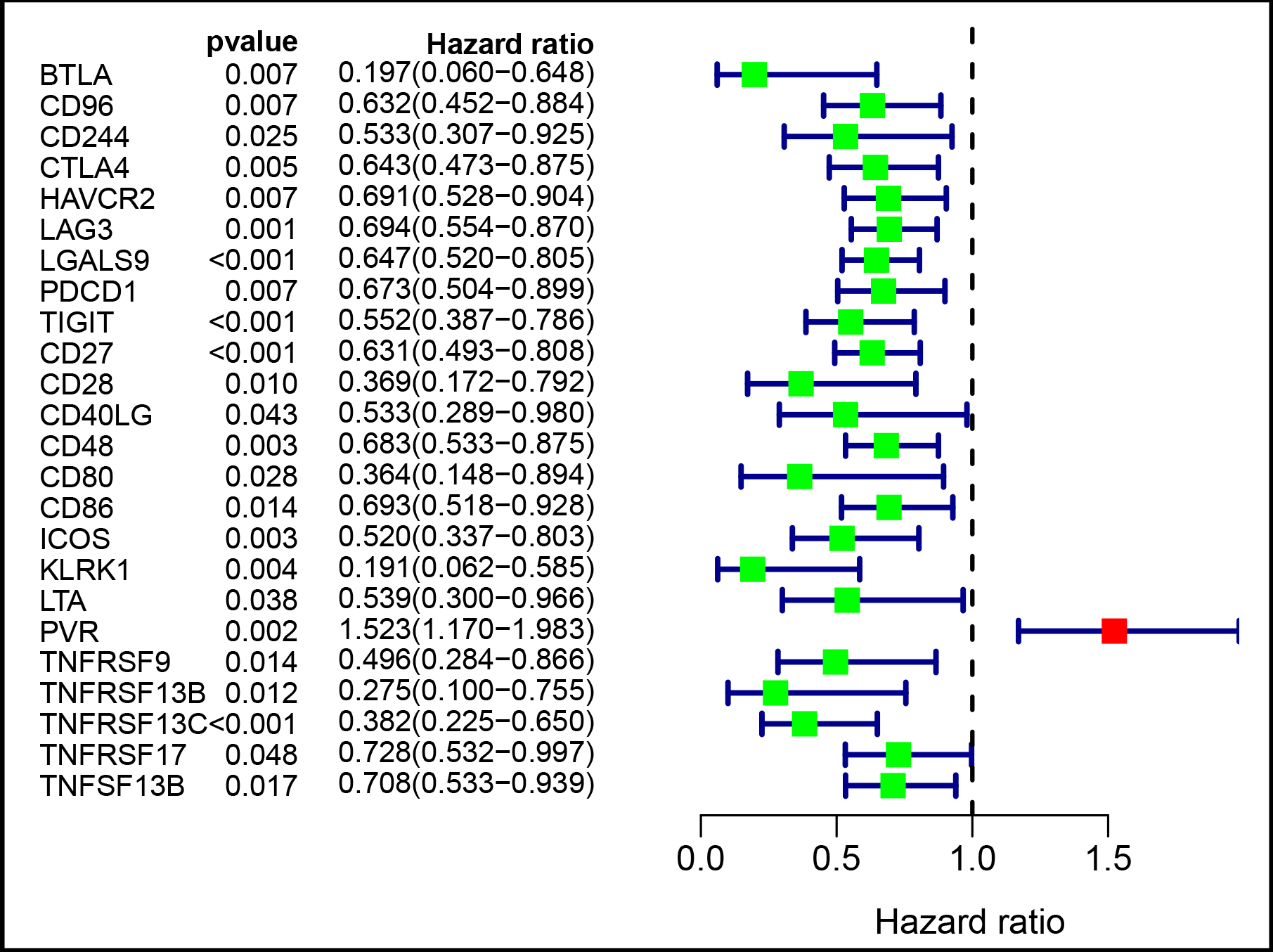

Supplement: Supplementary file 2 [file Image1.tif]
