# Supplementary material for: A novel ten-gene prognostic signature for cervical cancer based on CD79B-related immunomodulators
Source: Front Genet. 2022 Nov 2;13:933798. doi: 10.3389/fgene.2022.933798 (PMC9666757; doi:10.3389/fgene.2022.933798)
Supplement: Supplementary file 3 [file Table1.doc]

**Supplementary Table 1. Cervical cancer patient characteristics from TCGA database.**

| **Characteristic** | **Subtype** | **Overall** |
| --- | --- | --- |
| **Total,n** |  | 306 |
| **Age, median (IQR)** |  | 46 (38, 56) |
| **T stage, n (%)** | T1 | 140 (57.6%) |
|  | T2 | 72 (29.6%) |
|  | T3 | 21 (8.6%) |
|  | T4 | 10 (4.1%) |
| **N stage, n (%)** | N0 | 134 (68.7%) |
|  | N1 | 61 (31.3%) |
| **M stage, n (%)** | M0 | 116 (91.3%) |
|  | M1 | 11 (8.7%) |
| **Clinical stage, n (%)** | Stage I | 162 (54.2%) |
|  | Stage II | 69 (23.1%) |
|  | Stage III | 46 (15.4%) |
|  | Stage IV | 22 (7.4%) |
| **Radiation therapy, n (%)** | No | 122 (39.9%) |
|  | Yes | 184 (60.1%) |
| **Primary therapy outcome, n (%)** | PD | 23 (10.5%) |
|  | SD | 6 (2.7%) |
|  | PR | 8 (3.7%) |
|  | CR | 182 (83.1%) |
| **Race, n (%)** | Asian | 20 (7.7%) |
|  | Black or African American | 31 (11.9%) |
|  | White | 210 (80.5%) |
| **Age, n (%)** | <=50 | 188 (61.4%) |
|  | >50 | 118 (38.6%) |
| **BMI, n (%)** | <=25 | 100 (38.5%) |
|  | >25 | 160 (61.5%) |
| **Histological type, n (%)** | Adenosquamous | 53 (17.3%) |
|  | Squamous cell carcinoma | 253 (82.7%) |
| **Histologic grade, n (%)** | G1 | 19 (6.9%) |
|  | G2 | 135 (49.3%) |
|  | G3 | 119 (43.4%) |
|  | G4 | 1 (0.4%) |
| **Menopause status, n (%)** | Pre | 126 (54.1%) |
|  | Peri | 25 (10.7%) |
|  | Post | 82 (35.2%) |
| **Keratinizing squamous cell carcinoma present, n (%)** | No | 120 (39.2%) |
|  | Yes | 186 (60.8%) |
| **DSS event, n (%)** | Alive | 247 (81.8%) |
|  | Dead | 55 (18.2%) |
| **PFI event, n (%)** | Alive | 234 (76.5%) |
|  | Dead | 72 (23.5%) |
| **OS event, n (%)** | Alive | 234 (76.5%) |
|  | Dead | 72 (23.5%) |

**Abbreviations:** IQR, interquartile range; M,distant metastasis; N , lymph node metastasis; T, tumor depth; CR, complete response; PR, partial response; SD, stable disease; PD, progressive disease; BMI, body mass index;DSS, disease-specific disease; PFI, progression-free interval.
