# Supplementary material for: A novel ten-gene prognostic signature for cervical cancer based on CD79B-related immunomodulators
Source: Front Genet. 2022 Nov 2;13:933798. doi: 10.3389/fgene.2022.933798 (PMC9666757; doi:10.3389/fgene.2022.933798)
Supplement: Supplementary file 4 [file Table2.doc]

Supplementary Table 2 | Univariate cox regression analysis showing associations between 146 DEGs and OS in cervical cancer.

| **Gene** | **Univariate cox regression analysis** | | | |
| --- | --- | --- | --- | --- |
|  | **HR** | **HR.95L** | **HR.95H** | ***P*-value** |
| BTNL8 | 0.316866236 | 0.188526254 | 0.532574163 | 1.44E-05 |
| CCR7 | 0.393872359 | 0.239419937 | 0.647963729 | 0.000244081 |
| SPIB | 0.399227021 | 0.242391406 | 0.657540697 | 0.000310045 |
| LY9 | 0.419618392 | 0.257546385 | 0.683681095 | 0.000488895 |
| CD6 | 0.421298375 | 0.258280767 | 0.687206885 | 0.000535021 |
| CD27 | 0.422710647 | 0.25834257 | 0.691656397 | 0.00060937 |
| CD1E | 0.436100774 | 0.268435798 | 0.708489281 | 0.000802647 |
| JAK3 | 0.442871572 | 0.270685071 | 0.724588278 | 0.001185006 |
| GRAP2 | 0.449996305 | 0.276278569 | 0.732943838 | 0.001335618 |
| SLAMF6 | 0.451328071 | 0.27586398 | 0.738396612 | 0.001538065 |
| SELPLG | 0.45597464 | 0.27851809 | 0.746496834 | 0.001793892 |
| ZNF831 | 0.462905046 | 0.285042619 | 0.751751027 | 0.001849528 |
| CD5 | 0.468025487 | 0.288626553 | 0.758931756 | 0.002081105 |
| CD79A | 0.469013979 | 0.28910048 | 0.76089155 | 0.002163087 |
| BIN2 | 0.474485211 | 0.294328259 | 0.764915392 | 0.002214215 |
| LILRA4 | 0.47242671 | 0.292023401 | 0.764277779 | 0.002248739 |
| FOXP3 | 0.478558585 | 0.297238286 | 0.770487282 | 0.002421388 |
| CD3G | 0.47359283 | 0.29206762 | 0.767939181 | 0.00244047 |
| PLA2G2D | 0.480722015 | 0.298954317 | 0.773006584 | 0.002508277 |
| CD1C | 0.476405056 | 0.294306471 | 0.77117495 | 0.002550118 |
| SELL | 0.476224138 | 0.293533537 | 0.77261846 | 0.002657211 |
| TIGIT | 0.480057008 | 0.296009521 | 0.778538235 | 0.002932484 |
| CD79B | 0.477402528 | 0.293243244 | 0.777215429 | 0.002943646 |
| UBASH3A | 0.483044236 | 0.297849893 | 0.783386998 | 0.003182376 |
| CCR2 | 0.486802758 | 0.301225764 | 0.786708686 | 0.00328712 |
| CHIT1 | 0.491887497 | 0.305554347 | 0.79185033 | 0.003492598 |
| SLAMF1 | 0.486728768 | 0.300136133 | 0.7893248 | 0.003511162 |
| POU2AF1 | 0.487520772 | 0.299420612 | 0.793788048 | 0.003871202 |
| TBC1D10C | 0.497408184 | 0.307642958 | 0.804227416 | 0.004389491 |
| CD1B | 0.501907922 | 0.311484157 | 0.808745989 | 0.004625128 |
| GNG8 | 0.495443608 | 0.304263237 | 0.806750008 | 0.004754511 |
| SIT1 | 0.503875063 | 0.312716189 | 0.811886586 | 0.00485974 |
| P2RY8 | 0.502457593 | 0.309862347 | 0.814760604 | 0.005260887 |
| PSTPIP1 | 0.506209972 | 0.3133201 | 0.817849017 | 0.005411098 |
| FCMR | 0.501552558 | 0.307909494 | 0.81697698 | 0.00557126 |
| PPP1R16B | 0.506814486 | 0.312677553 | 0.821488208 | 0.005816603 |
| P2RY13 | 0.51047779 | 0.315889908 | 0.824931621 | 0.00603483 |
| PYHIN1 | 0.518463983 | 0.320934002 | 0.837570654 | 0.00726882 |
| PRKCB | 0.518811296 | 0.319986478 | 0.841176673 | 0.00778137 |
| CXCR6 | 0.52622042 | 0.327462028 | 0.845618442 | 0.007981962 |
| PTGDS | 0.525264842 | 0.326067035 | 0.846154698 | 0.008129022 |
| PLD4 | 0.527255672 | 0.327874127 | 0.847881919 | 0.008271762 |
| P2RY10 | 0.52487447 | 0.32498794 | 0.847702869 | 0.008401197 |
| CD300LF | 0.529074726 | 0.329415606 | 0.849747432 | 0.008451721 |
| RHOH | 0.525449395 | 0.325299226 | 0.848747996 | 0.008531555 |
| CD3D | 0.524276206 | 0.323302832 | 0.850179808 | 0.008844527 |
| RIPOR2 | 0.528051811 | 0.325636714 | 0.856287706 | 0.009625359 |
| KLRD1 | 0.530879876 | 0.328682044 | 0.857465284 | 0.009636912 |
| SCUBE1 | 0.531095311 | 0.328811526 | 0.857823427 | 0.009685221 |
| CST7 | 0.5315261 | 0.329050995 | 0.858590309 | 0.009791432 |
| PLA2G7 | 0.536365153 | 0.333854923 | 0.861714348 | 0.010017263 |
| SCML4 | 0.53223979 | 0.329304952 | 0.86023363 | 0.010036682 |
| GZMM | 0.528021418 | 0.324342494 | 0.85960558 | 0.010217326 |
| AMPD1 | 0.530835374 | 0.327285004 | 0.860981074 | 0.010270643 |
| MS4A6A | 0.538480386 | 0.335228886 | 0.86496462 | 0.010470657 |
| CD2 | 0.537505472 | 0.332610405 | 0.868620247 | 0.011240647 |
| ABCD2 | 0.544919364 | 0.339140581 | 0.875557598 | 0.012099938 |
| IL12RB1 | 0.544024046 | 0.337514082 | 0.876888339 | 0.012442428 |
| BLK | 0.551381399 | 0.343221697 | 0.885787377 | 0.013839976 |
| ASGR2 | 0.545675854 | 0.33655309 | 0.884740466 | 0.014025058 |
| SLA2 | 0.550587692 | 0.340872495 | 0.889326102 | 0.014711011 |
| HLA-DQA1 | 0.558153414 | 0.348118293 | 0.894911986 | 0.015481176 |
| JCHAIN | 0.557660247 | 0.346289538 | 0.898048937 | 0.016292862 |
| SP140 | 0.562794953 | 0.350438736 | 0.903833185 | 0.017393189 |
| BTLA | 0.559324724 | 0.346276326 | 0.90345231 | 0.017549738 |
| GPR171 | 0.557216085 | 0.343647992 | 0.903511072 | 0.017719853 |
| TTC24 | 0.560525378 | 0.347007278 | 0.90542395 | 0.017979381 |
| MPEG1 | 0.566958067 | 0.353736545 | 0.908702971 | 0.018386877 |
| RASAL3 | 0.565981892 | 0.35214981 | 0.909656893 | 0.018719221 |
| CYSLTR2 | 0.566965976 | 0.353009267 | 0.910600506 | 0.018906717 |
| TCL1A | 0.566166183 | 0.35148072 | 0.911982159 | 0.019348237 |
| PRAM1 | 0.56806484 | 0.353358604 | 0.913229956 | 0.019559712 |
| PILRA | 0.571084587 | 0.356344197 | 0.915231981 | 0.019908891 |
| P2RX1 | 0.567869699 | 0.352454417 | 0.914943832 | 0.020059092 |
| CYTIP | 0.566694824 | 0.350714079 | 0.915683295 | 0.02035444 |
| LST1 | 0.5697911 | 0.353688616 | 0.917931432 | 0.020781452 |
| CASQ1 | 0.572433131 | 0.356474072 | 0.919224468 | 0.020971439 |
| TESPA1 | 0.570709046 | 0.35448703 | 0.9188173 | 0.020974769 |
| RASGRP2 | 0.569712775 | 0.352700381 | 0.920250342 | 0.021467147 |
| IGSF6 | 0.573831767 | 0.357377535 | 0.921386668 | 0.021513969 |
| TNFSF8 | 0.5744744 | 0.357803048 | 0.922353337 | 0.021758876 |
| TREM2 | 0.576405952 | 0.35981736 | 0.923367959 | 0.021929851 |
| VCAM1 | 0.573932824 | 0.356418131 | 0.924192285 | 0.022354176 |
| CD3E | 0.578022033 | 0.359968664 | 0.928162654 | 0.023299926 |
| CXCL9 | 0.579417253 | 0.361544931 | 0.928582656 | 0.023336416 |
| PTPN7 | 0.580447886 | 0.361200202 | 0.932778405 | 0.024609243 |
| IL16 | 0.581809185 | 0.362250048 | 0.934442741 | 0.025061895 |
| RASSF4 | 0.583461378 | 0.36413026 | 0.934904941 | 0.02510492 |
| LAPTM5 | 0.577261246 | 0.356894385 | 0.933695123 | 0.025116962 |
| SELP | 0.580250548 | 0.360343806 | 0.934359612 | 0.025137288 |
| SNX20 | 0.583932562 | 0.363651399 | 0.937648635 | 0.025987247 |
| C20orf141 | 1.722028229 | 1.066318615 | 2.780952314 | 0.026246367 |
| SAMSN1 | 0.582978545 | 0.36204177 | 0.938742466 | 0.026416117 |
| GPR183 | 0.584311969 | 0.363558708 | 0.939106862 | 0.026454395 |
| KLHL6 | 0.587829791 | 0.366656309 | 0.942418977 | 0.027368542 |
| CD48 | 0.586997473 | 0.365487185 | 0.94275818 | 0.027537804 |
| ITK | 0.583615325 | 0.361367439 | 0.942549913 | 0.02767344 |
| TMEM273 | 0.587447106 | 0.365798366 | 0.943399792 | 0.027733357 |
| CD1A | 0.586679555 | 0.364706357 | 0.9437535 | 0.027903598 |
| TREML1 | 0.589545643 | 0.367860914 | 0.944824663 | 0.028105026 |
| CXCR3 | 0.588184487 | 0.366089408 | 0.945017756 | 0.028255461 |
| CLNK | 0.589087366 | 0.36674512 | 0.946226425 | 0.028628758 |
| KCNJ10 | 0.588025107 | 0.365192357 | 0.946825748 | 0.028904557 |
| CD8A | 0.589487561 | 0.366901593 | 0.947108411 | 0.028919054 |
| JAML | 0.588390473 | 0.365469946 | 0.947282678 | 0.029045717 |
| IRF4 | 0.592538353 | 0.369784198 | 0.949477294 | 0.029594678 |
| KCNA3 | 0.591211605 | 0.368120411 | 0.949502259 | 0.029680156 |
| JAKMIP1 | 0.591580651 | 0.36841541 | 0.949926785 | 0.02981323 |
| NFAM1 | 0.593636404 | 0.37040414 | 0.95140454 | 0.0302374 |
| CLEC9A | 0.594225986 | 0.370550537 | 0.952918663 | 0.030764522 |
| TLR10 | 0.592350741 | 0.367979906 | 0.953528698 | 0.031093608 |
| WAS | 0.594460031 | 0.3699382 | 0.95524801 | 0.031622296 |
| RAB33A | 0.594860214 | 0.370392487 | 0.955361369 | 0.031643663 |
| LAX1 | 0.596274938 | 0.372009647 | 0.955738123 | 0.031710698 |
| NCF1 | 0.59588965 | 0.37146179 | 0.955911173 | 0.031796594 |
| BTK | 0.598068967 | 0.373227489 | 0.958360518 | 0.032617997 |
| MS4A4E | 0.600137266 | 0.37529697 | 0.959679312 | 0.033023705 |
| CXorf65 | 0.597912171 | 0.372556977 | 0.959581987 | 0.033097154 |
| CD28 | 0.59797821 | 0.372240974 | 0.960608756 | 0.033492025 |
| TIMD4 | 0.596107544 | 0.369877787 | 0.960707066 | 0.033620998 |
| THEMIS | 0.601189777 | 0.375017331 | 0.963766521 | 0.034580592 |
| IGLL5 | 0.601816368 | 0.374828277 | 0.966263654 | 0.035551188 |
| CYBB | 0.604239537 | 0.377100034 | 0.968192482 | 0.036229392 |
| CASS4 | 0.60290491 | 0.374377614 | 0.970929663 | 0.037405411 |
| ADA2 | 0.606373317 | 0.378362722 | 0.97178865 | 0.03762793 |
| CSF2RB | 0.603471491 | 0.374710873 | 0.971890239 | 0.03778017 |
| SIGLEC1 | 0.608191111 | 0.380410352 | 0.972361625 | 0.037798594 |
| SIGLEC10 | 0.608687712 | 0.380435881 | 0.973884823 | 0.038422576 |
| ICOS | 0.607681775 | 0.379194002 | 0.973847522 | 0.038443065 |
| CCL5 | 0.604531366 | 0.375347081 | 0.973653964 | 0.038474698 |
| CLECL1 | 0.606524024 | 0.377610151 | 0.974209489 | 0.038636965 |
| TBX21 | 0.6069443 | 0.377977669 | 0.974611501 | 0.038791759 |
| GMFG | 0.609946382 | 0.380286314 | 0.978301285 | 0.040269578 |
| SH2D1A | 0.611455692 | 0.381367856 | 0.980360712 | 0.041120465 |
| FERMT3 | 0.613344403 | 0.382809653 | 0.982711261 | 0.042105427 |
| CD72 | 0.614417084 | 0.383830839 | 0.983527934 | 0.042442274 |
| CD37 | 0.615503942 | 0.384682395 | 0.984825685 | 0.042998475 |
| CLEC10A | 0.618221638 | 0.386570097 | 0.988690012 | 0.044702407 |
| TRAT1 | 0.616301918 | 0.383689935 | 0.989934892 | 0.045306015 |
| GZMK | 0.618860139 | 0.386188372 | 0.99171259 | 0.046091627 |
| CTLA4 | 0.620117847 | 0.387729318 | 0.991790214 | 0.046111597 |
| ARHGAP9 | 0.620225643 | 0.386287781 | 0.995837475 | 0.048015648 |
| AIF1 | 0.621261993 | 0.386930465 | 0.997508593 | 0.048805214 |
| GPR18 | 0.621533917 | 0.387128793 | 0.997870521 | 0.048977302 |
| TNFRSF8 | 0.624707135 | 0.390714544 | 0.998834086 | 0.049433218 |
| CD40LG | 0.624733941 | 0.390506344 | 0.999452384 | 0.049733523 |

Abbreviations:DGEs,differentially expressed genes; OS, overall survival; HR,hazard ratio.
